# Deep learning-quantified body composition from positron emission tomography/computed tomography and cardiovascular outcomes: a multicentre study

**DOI:** 10.1093/eurheartj/ehaf131

**Published:** 2025-03-30

**Authors:** Robert J H Miller, Jirong Yi, Aakash Shanbhag, Anna Marcinkiewicz, Krishna K Patel, Mark Lemley, Giselle Ramirez, Jolien Geers, Panithaya Chareonthaitawee, Samuel Wopperer, Daniel S Berman, Marcelo Di Carli, Damini Dey, Piotr J Slomka

**Affiliations:** Departments of Medicine (Division of Artificial Intelligence in Medicine), Imaging and Biomedical Sciences, Cedars-Sinai Medical Center, 6500 Wilshire Blvd, Suite 420, Los Angeles, CA 90048, USA; Department of Cardiac Sciences, University of Calgary, Calgary, AB, Canada; Departments of Medicine (Division of Artificial Intelligence in Medicine), Imaging and Biomedical Sciences, Cedars-Sinai Medical Center, 6500 Wilshire Blvd, Suite 420, Los Angeles, CA 90048, USA; Departments of Medicine (Division of Artificial Intelligence in Medicine), Imaging and Biomedical Sciences, Cedars-Sinai Medical Center, 6500 Wilshire Blvd, Suite 420, Los Angeles, CA 90048, USA; Signal and Image Processing Institute, Ming Hsieh Department of Electrical and Computer Engineering, University of Southern California, Los Angeles, CA, USA; Departments of Medicine (Division of Artificial Intelligence in Medicine), Imaging and Biomedical Sciences, Cedars-Sinai Medical Center, 6500 Wilshire Blvd, Suite 420, Los Angeles, CA 90048, USA; Center of Radiological Diagnostics, National Medical Institute of the Ministry of the Interior and Administration, Warsaw, Poland; Department of Medicine (Cardiology) and Population Health Science and Policy, Icahn School of Medicine at Mount Sinai, New York, NY, USA; Departments of Medicine (Division of Artificial Intelligence in Medicine), Imaging and Biomedical Sciences, Cedars-Sinai Medical Center, 6500 Wilshire Blvd, Suite 420, Los Angeles, CA 90048, USA; Departments of Medicine (Division of Artificial Intelligence in Medicine), Imaging and Biomedical Sciences, Cedars-Sinai Medical Center, 6500 Wilshire Blvd, Suite 420, Los Angeles, CA 90048, USA; Departments of Medicine (Division of Artificial Intelligence in Medicine), Imaging and Biomedical Sciences, Cedars-Sinai Medical Center, 6500 Wilshire Blvd, Suite 420, Los Angeles, CA 90048, USA; Department of Cardiology, Centrum voor Hart-en Vaatziekten (CHVZ), Universitair Ziekenhuis Brussel (UZ Brussel), Vrije Universiteit Brussel (VUB), Brussels, Belgium; Department of Cardiovascular Medicine, Mayo Clinic, Rochester, MN, USA; Department of Cardiovascular Medicine, Mayo Clinic, Rochester, MN, USA; Departments of Medicine (Division of Artificial Intelligence in Medicine), Imaging and Biomedical Sciences, Cedars-Sinai Medical Center, 6500 Wilshire Blvd, Suite 420, Los Angeles, CA 90048, USA; Division of Nuclear Medicine and Molecular Imaging, Department of Radiology, Brigham and Women’s Hospital, Boston, MA, USA; Departments of Medicine (Division of Artificial Intelligence in Medicine), Imaging and Biomedical Sciences, Cedars-Sinai Medical Center, 6500 Wilshire Blvd, Suite 420, Los Angeles, CA 90048, USA; Departments of Medicine (Division of Artificial Intelligence in Medicine), Imaging and Biomedical Sciences, Cedars-Sinai Medical Center, 6500 Wilshire Blvd, Suite 420, Los Angeles, CA 90048, USA

**Keywords:** Body composition, Positron emission tomography, Deep learning, Risk Stratification

## Abstract

**Background and Aims:**

Positron emission tomography (PET)/computed tomography (CT) myocardial perfusion imaging (MPI) is a vital diagnostic tool, especially in patients with cardiometabolic syndrome. Low-dose CT scans are routinely performed with PET for attenuation correction and potentially contain valuable data about body tissue composition. Deep learning and image processing were combined to automatically quantify skeletal muscle (SM), bone and adipose tissue from these scans and then evaluate their associations with death or myocardial infarction (MI).

**Methods:**

In PET MPI from three sites, deep learning quantified SM, bone, epicardial adipose tissue (EAT), subcutaneous adipose tissue (SAT), visceral adipose tissue (VAT), and intermuscular adipose tissue (IMAT). Sex-specific thresholds for abnormal values were established. Associations with death or MI were evaluated using unadjusted and multivariable models adjusted for clinical and imaging factors.

**Results:**

This study included 10 085 patients, with median age 68 (interquartile range 59–76) and 5767 (57%) male. Body tissue segmentations were completed in 102 ± 4 s. Higher VAT density was associated with an increased risk of death or MI in both unadjusted [hazard ratio (HR) 1.40, 95% confidence interval (CI) 1.37–1.43] and adjusted (HR 1.24, 95% CI 1.19–1.28) analyses, with similar findings for IMAT, SAT, and EAT. Patients with elevated VAT density and reduced myocardial flow reserve had a significantly increased risk of death or MI (adjusted HR 2.49, 95% CI 2.23–2.77).

**Conclusions:**

Volumetric body tissue composition can be obtained rapidly and automatically from standard cardiac PET/CT. This new information provides a detailed, quantitative assessment of sarcopenia and cardiometabolic health for physicians.

## Structured Graphical Abstract

**Structured Graphical Abstract:**
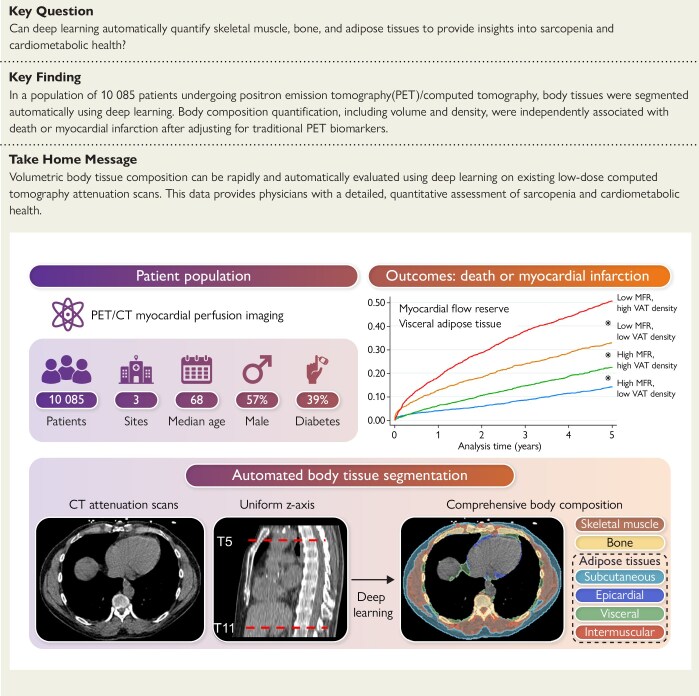
Overview of the study design. Automated body tissue segmentation of computed tomography (CT) attenuation scans by deep learning. This study evaluated correlations between variables and associations with outcomes. MFR, myocardial flow reserve; PET, positron emission tomography; VAT, visceral adipose tissue.

**See the editorial comment for this article ‘Expanding CAD assessment beyond coronary arteries: can body composition offer valuable insights?’, by M. Al-Mallah and M. Al Rifai, https://doi.org/10.1093/eurheartj/ehaf143.**

## Introduction

Positron emission tomography (PET) myocardial perfusion imaging (MPI) is an essential diagnostic tool for assessing patients with known or suspected coronary artery disease (CAD).^1^ Positron emission tomography MPI uniquely integrates assessment of both epicardial coronary arteries and microvasculature by capturing relative perfusion and measuring absolute myocardial blood flow (MBF), thereby providing superior diagnostic accuracy for CAD compared to other modalities.^2,3^ Positron emission tomography MPI also serves as a key predictor of cardiac events through markers such as relative perfusion, left ventricular function, and quantitative myocardial flow metrics.^4–9^ Due to these unique strengths of PET MPI, patients with high risk of epicardial and microvascular disease, such as those with cardiometabolic syndrome, are increasingly referred for PET MPI.^10^ This, in combination with rising rates of cardiometabolic syndrome and an aging population with rising rates of frailty,^11,12^ necessitates consideration of factors beyond perfusion alone to help guide management.

To complement relative and absolute perfusion assessments, anatomic information is frequently evaluated from computed tomography (CT) attenuation correction (AC) imaging. Coronary artery calcium (CAC) from CTAC can provide independent prognostic information.^13–16^ However, the CTAC scans contain a wealth of information regarding body tissue composition, which is currently not utilized clinically but could potentially provide clinicians with critical insights. For example, epicardial adipose tissue (EAT) volume and density are valuable measures of metabolic health and inflammation,^17,18^ with similar information potentially available from visceral adipose tissue (VAT) quantification.^19^ This information is particularly relevant for patients with cardiometabolic syndrome. Skeletal muscle (SM) measurements can provide quantitative measures of sarcopenia, a valuable marker of risk. Sarcopenia is also inter-related with bone mineral density.^20^ In this study, we evaluated an automated pipeline designed to segment subcutaneous adipose tissue (SAT), EAT, VAT, intermuscular adipose tissue (IMAT), bone, and SM to provide quantitative measures of sarcopenia and cardiometabolic health. We then evaluated their independent prognostic utility after adjusting for existing markers of risk from PET/CT MPI including CAC, relative perfusion, and myocardial flow reserve (MFR).

## Methods

### Patient population

An overview of our study designed is shown in *Structured Graphic Abstract*. We included consecutive patients from three sites who underwent PET MPI with CTAC imaging between 2007 and 2020 at Brigham and Women’s Hospital, between 2010 and 2018 at Cedars-Sinai Medical Center, and between 2014 and 2022 at Mayo Clinic. Patients with CT scans that did not capture the entire range between T5 and T11 vertebrae (*n* = 1269) were excluded. The proportion of missing data as a function of vertebral height is shown in Supplementary data online, *Figure S1*. In total, 10 085 patients were included in the analysis. This study was approved by the institutional review boards at each participating site, with approval of the overall study provided by the Cedars-Sinai Medical Center review board.

### Clinical variables

Clinical information was collected at the time of imaging and included: age, sex, body mass index (BMI), family history of CAD, smoking status, previous myocardial infarction (MI), previous revascularization, and history of hypertension, diabetes, dyslipidaemia, heart failure, and cancer. Each site collected this information using different, site-specific procedures. Medical history, age, and sex were complete for all patients. Body mass index was not available for 29 patients (0.3%) and was imputed with population median if missing.

### Positron emission tomography imaging and quantification

Patients underwent rest and stress PET MPI in accordance with clinical guidelines.^3^ Patients were imaged with either rubidium-82 (*n* = 6447) or N13-ammonia (*n* = 3638). Early dynamic acquisitions were utilized to quantify stress and rest MBF, with MFR calculated as stress MBF/rest MBF.^21^ Stress and rest relative perfusion was quantified using total perfusion deficit (TPD).^22^ Stress and rest left ventricular ejection fraction were quantified from gated images, with change in ejection fraction calculated as the difference between the two values. All PET image quantification was performed at the core laboratory blinded to outcomes and body composition quantification with dedicated software (QPET, Cedars-Sinai Medical Center, Los Angeles, CA).^22,23^

### Computed tomography attenuation correction image acquisition

Computed tomography attenuation correction scans were acquired according to site-specific protocols as outlined in Supplementary data online, *Table S1*. At Brigham and Women’s Hospital, CTAC scans were acquired with tube voltage 80–140 kVp, tube current 10–300 mAs, and slice thickness of 2.5–5 mm. At Cedars-Sinai Medical Center, CTAC scans were acquired with tube voltage 100 kVp, tube current 11–13 mAs, and slice thickness of 3 mm. At Mayo Clinic, CTAC scans were acquired with tube voltage 120–140 kVp, tube current 20–180 mAs, and slice thickness of 3.3 mm.

### Computed tomography attenuation correction segmentation

Our approach for body composition quantification was performed using deep learning models as outlined in Supplementary data online, *Figure S2*. In brief, CAC and EAT segmentations are completed using a convolutional long short-term memory model as previously described.^15,17,24^ Body composition was completed using image processing based on segmented organs from a nnUNet.^25,26^ First, the body was separated into intrathoracic (not including bones) and extrathoracic components (including bones) using a convex hull-based algorithm to encompass the intrathoracic organs. Tissue types were differentiated using Hounsfield unit (HU) ranges, with adipose tissue defined as HU −190 to −30, muscle defined as HU −29–150, and bone as between +151 and +1200 HU. Adipose tissue within the intrathoracic space was considered VAT, unless previously segmented as EAT. Four tissues (SAT, SM, IMAT, and bone) were segmented in the extrathoracic space. Extrathoracic tissue with muscle density was attributed to SM, and bone density was attributed to bone. Extrathoracic tissue with adipose density was attributed to SAT if it was in contact with skin and otherwise was classified as IMAT. To reduce the effects of variations in scanned body regions, we limited segmentation to areas most frequently captured on CT imaging (T5 to T11 from automatic segmentations) as shown in Supplementary data online, *Figure S1*. Limiting the quantified thoracic volume leads to standardized measurements which can be comparable across sites.

### Volumetric body composition quantification

After segmentation, we quantified each body tissue component. Volumes were indexed to body surface area (calculated using the DuBois formula^27^) and quantified as cm^3^/m^2^. However, we also performed sensitivity analyses where volumes were indexed to total thoracic volume. Mean density (in HU) was also quantified for each body tissue component using the entire volume of tissue. Since existing cut-offs were not available for the proposed volumetric analysis, we determined optimal sex-specific cut-offs for abnormality using the Youden index. This approach maximizes the sensitivity and specificity for identifying death or MI. We did not utilize an approach based on mean and standard deviation since patients undergoing PET MPI represent a highly selected population.

### Outcome

The primary outcome was incidence of death or MI. Death was ascertained from the National Death Index which is considered to be the most complete source of information available, typically capturing more events than the social security administrative death master file^28^ or local electronic medical records.^29^ Incidence of MI was determined from electronic medical records, with all potential events adjudicated by an experienced physician based on available clinical, laboratory, imaging, and invasive angiography information. We also evaluated associations with each event separately (death, MI) as well as associations with cardiovascular death. Cardiovascular death was adjudicated using available clinical information and included sudden cardiac death and death due to MI, heart failure, stroke, cardiovascular procedure, cardiovascular haemorrhage, and other cardiovascular causes (e.g. pulmonary embolism or peripheral arterial disease).^30^

### Statistical analysis

Continuous variables were summarized as mean [standard deviation (SD)] if normally distributed and compared using a Student’s *t*-test. Continuous variables that were not normally distributed were summarized as median [interquartile range (IQR)] and compared using a Mann–Whitney *U* test. The correlations between body tissue components were assessed using correlation coefficients. Associations with death or MI were assessed using unadjusted and multivariable models. Multivariable models included age, sex, BMI, medical history (previous MI, previous revascularization, and history of hypertension, diabetes, dyslipidaemia, heart failure, and cancer), CAC, stress TPD, stress left ventricular ejection fraction, change in ejection fraction, MFR, and revascularization within 90 days. Coronary artery calcium was modelled as a categorical variable in multivariable models given the importance of CAC of zero.^31^ Total perfusion deficit, stress left ventricular ejection fraction, and MFR were log transformed since they were not normally distributed. Associations were assessed overall as well as stratified by sex and age. Improvement in model fit was assessed with likelihood ratio (LR) χ^2^ and compared using the LR tests. Associations with MI were modelled using a competing risk analysis, with death as the competing risk. The proportional hazards assumption was assessed using Schoenfeld residuals and log–log plots. All statistical tests were two-sided, and a *P*-value of <.05 was considered statistically significant. All analyses were performed using Stata/IC version 13.1 (StataCorp, College Station, TX, USA) and R Studio (version 4.3.2) including the following opensource packages: corrplot and PredictABEL.

## Results

### Patient population

**Figure 1:**
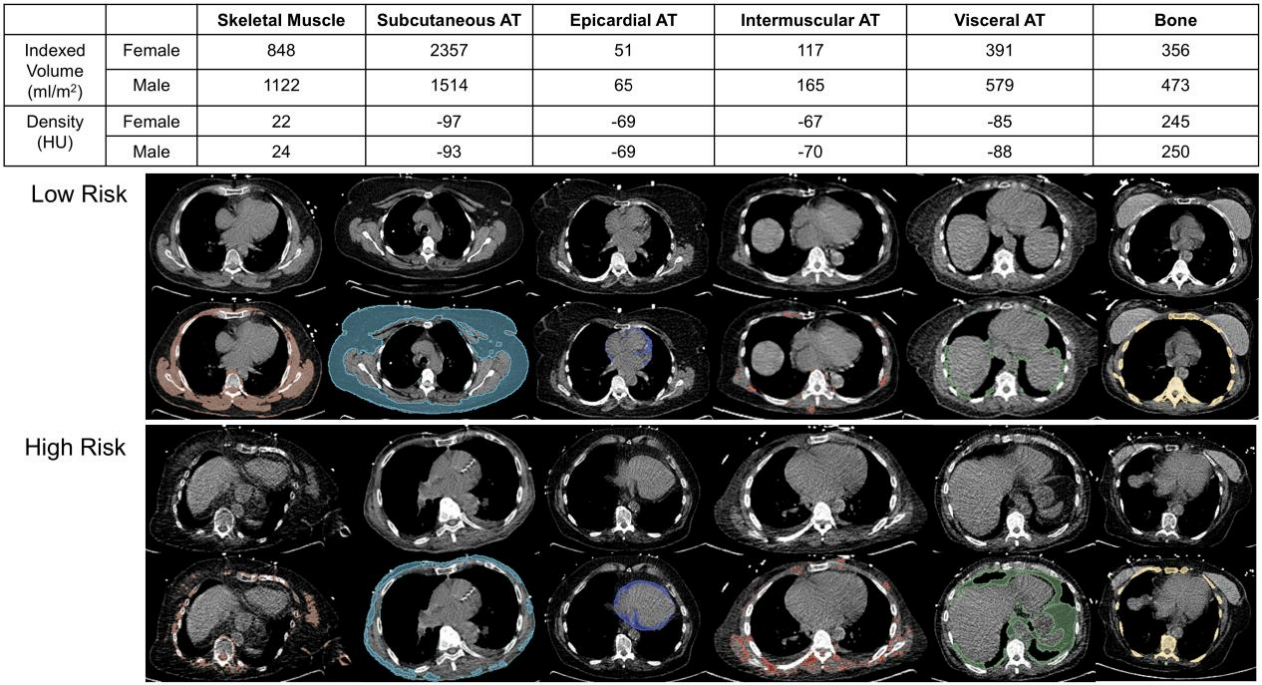
Sex-specific thresholds for abnormal indexed volumes and density. Skeletal muscle and bone volume and density were associated with lower risk above threshold. Subcutaneous adipose tissue (AT) volume above threshold was associated with reduced risk, while density above threshold was associated with increased risk. Volume or density above threshold was associated with increased risk for epicardial, intermuscular, and visceral AT. Examples of cases with low risk (top) and high-risk metrics (bottom). Segmentations are shown in an overlay. Skeletal muscle is shown in light brown, subcutaneous adipose tissue in light blue, epicardial adipose tissue in dark blue, intermuscular adipose tissue in dark brown, and visceral adipose tissue in green. AT, adipose tissue; HU, Hounsfield units

We included 10 085 patients, with median age 68 (IQR 59–76) and 5767 (57.2%) male. Patient characteristics and imaging findings stratified by SM and SAT volumes are shown in *Table 1*. The sex-specific thresholds for abnormal indexed tissue volumes and tissue density are shown in *Figure 1*. Patients with high SM volume were younger compared to patients with low volumes (median age 64 vs. 71, *P* < .001). Diabetes was more common in patients with high SAT volumes compared to patients with low SAT volumes (47.9% vs. 28.5%, *P* < .001). Patients with high SM and SAT volumes had the lowest CAC scores (median 68 vs. 112, 171, and 334; *P* < .001)

**Table 1:** Population characteristics stratified by skeletal muscle and subcutaneous adipose tissue volume

	Overall *n* = 10 085	High SM High SAT *n* = 2262	High SM Low SAT *n* = 2382	Low SM High SAT *n* = 3111	Low SM Low SAT *n* = 2330
Age, median (IQR)	68 (59, 76)	64 (55, 71)	65 (55, 73)	70 (62, 77)	74 (66, 81)
Male, *n* (%)	5767 (57.2%)	1312 (58.0%)	1480 (62.1%)	1656 (53.2%)	1319 (56.6%)
BMI (body mass index), median (IQR)	29.4 (25.5, 35.3)	33.8 (29.8, 39.6)	26.4 (23.7, 29.4)	34 (29.4, 40.8)	25.2 (22.8, 28)
Past medical history, *n* (%)					
Hypertension	7967 (79.0%)	1846 (81.6%)	1688 (70.9%)	2639 (84.8%)	1794 (77.0%)
Diabetes mellitus	3917 (38.8%)	1040 (46.0%)	702 (29.5%)	1535 (49.3%)	640 (27.5%)
Dyslipidaemia	6960 (69.0%)	1591 (70.3%)	1514 (63.6%)	2267 (72.9%)	1588 (68.2%)
Family history of coronary artery disease	2093 (20.6%)	501 (22.1%)	439 (18.4%)	718 (23.1%)	435 (18.7%)
Smoking	817 (8.1%)	188 (8.3%)	198 (8.3%)	275 (8.8%)	156 (6.7%)
Heart failure	1965 (19.5%)	377 (16.7%)	355 (14.9%)	716 (23.0%)	517 (22.2%)
Cancer	1938 (19.2%)	346 (15.3%)	333 (14.0%)	758 (24.4%)	501 (21.5%)
Past myocardial infarction	2074 (20.6%)	481 (21.3%)	492 (20.7%)	590 (19.0%)	511 (21.9%)
Previous revascularization	2911 (28.9%)	698 (30.9%)	670 (28.1%)	830 (26.7%)	713 (30.6%)
Imaging findings					
Deep learning CAC, median (IQR)	157 (0, 1011)	68 (0, 679)	112 (0, 887)	171 (0, 1013)	334 (18, 1430)
Stress TPD, median (IQR)	5.6 (2.0, 13.3)	5.5 (2.2, 12.4)	4.9 (1.6, 12.6)	5.6 (2.2, 12.9)	6.4 (2.1, 15.8)
Stress TPD > 5%, *n* (%)	5377 (53.3%)	1201 (53.1%)	1182 (49.6%)	1673 (53.8%)	1321 (56.7%)
Rest TPD, median (IQR)	1.2 (0.1, 5.1)	1.1 (0.1, 4.5)	0.9 (0.0, 4.7)	1.2 (0.1, 4.9)	1.5 (0.1, 6.4)
Stress EF, median (IQR)	66 (53, 74)	65 (55, 73)	65 (53, 73)	67 (54, 75)	65 (49, 74)
Change in EF, median (IQR)	2.1 (−1.1, 5.2)	2.2 (−1.1, 5.4)	2.8 (−0.6, 6.0)	1.5 (−1.5, 4.6)	2.2 (−1.1, 5.2)
Stress MBF, median (IQR)	2.08 (1.49, 2.85)	2.22 (1.57, 3.029)	2.23 (1.66, 2.90)	1.90 (1.35, 2.68)	2.03 (1.49, 2.76)
MFR, median (IQR)	2.19 (1.69, 2.78)	2.40 (1.81, 3.07)	2.33 (1.78, 2.99)	2.10 (1.67, 2.63)	2.04 (1.59, 2.55)
MFR < 2, *n* (%)	4025 (39.9%)	732 (32.4%)	825 (34.6%)	1362 (43.8%)	1106 (47.5%)
Rest MBF, median (IQR)	0.96 (0.72, 1.26)	0.95 (0.73, 1.24)	0.97 (0.74, 1.25)	0.92 (0.69, 1.24)	1.01 (0.77, 1.33)

CAC, coronary artery calcium; EF, ejection fraction; IQR, interquartile range; MBF, myocardial blood flow; MFR, myocardial flow reserve; TPD, total perfusion deficit.

### Body composition

Segmentations took a mean time of 102.1 ± 4.4 s. The distribution of tissue volume and density values are shown in Supplementary data online, *Figure S3*. The correlation between tissue components is shown in Supplementary data online, *Figure S4*. Skeletal muscle volume and density were positively correlated (r = 0.46, *P* < .001). While adipose tissue volumes were negatively correlated with density (SAT: r = −0.68, EAT: r = −0.46, VAT: r = −0.23; IMAT: r = −0.52, all *P* < .001). The distribution of tissue volume and age and tissue density and age are shown in Supplementary data online, *Figures S5* and *S6*. Skeletal muscle volume index and density decreased with age while bone density decreased with age.

Correlations between age, BMI, stress TPD, CAC, and MFR with body tissue composition are shown in Supplementary data online, *Table S2*. Only SM volume, SM density, bone density, and VAT density had significant correlations with MFR. Coronary artery calcium had positive correlations with EAT and VAT volumes, but a negative correlation with SAT volume. The relationship between patient classification of abnormal SM and VAT volume as well as VAT density are shown in Supplementary data online, *Figure S7*. Increasing age [adjusted odds ratio (OR) 1.89, 95% confidence interval (CI) 1.80–1.99, per SD increase], history of heart failure (adjusted OR 1.21, 95% CI 1.08–1.37), and history of cancer (adjusted OR 1.33, 95% CI 1.19–1.49) were independently associated with low SM volume index (see Supplementary data online, *Table S3*). Increasing age (adjusted OR 1.22, 95% CI 1.16–1.28, per SD increase), diabetes (adjusted OR 1.12, 95% CI 1.02–1.23), and heart failure (adjusted OR 1.34, 95% CI 1.19–1.50) were independently associated with high VAT density (see Supplementary data online, *Table S4*). Low SM volume (adjusted OR 0.74, 95% CI 0.68–0.81, per SD increase) and low SM density (adjusted OR 0.88, 95% CI 0.81–0.96, per SD increase) were associated with reduced MFR, with full results in Supplementary data online, *Table S5*.

### Clinical outcomes

**Figure 2:**
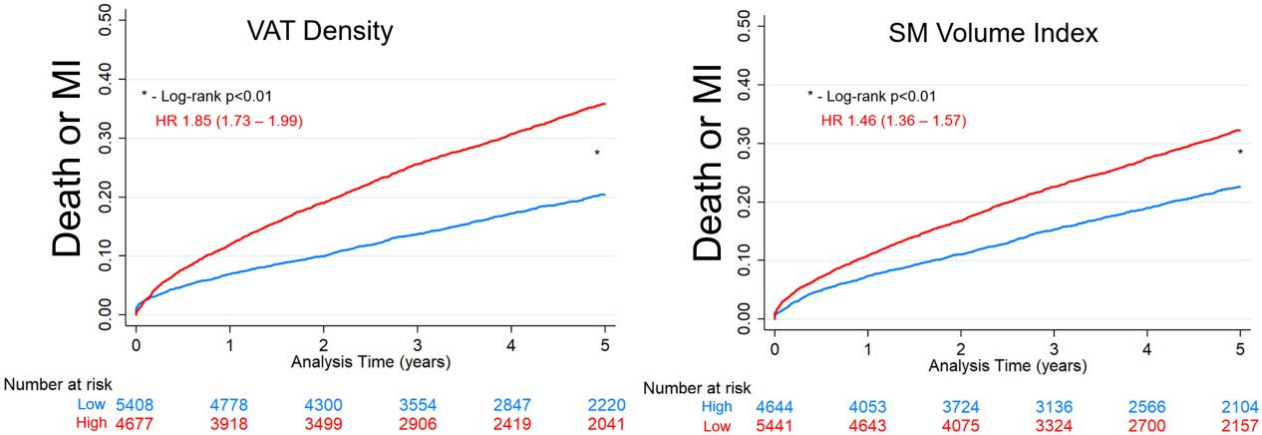
Unadjusted Kaplan–Meier curves as a function of visceral adipose tissue (VAT) density and skeletal muscle (SM) volume index. Hazard ratios (HRs) with 95% confidence intervals are shown. MI, myocardial infarction

During a median follow-up of 4.2 years (IQR 2.2, 7.0), 3322 (32.9%) patients experienced death or MI. The first event was MI in 690 patients, and 2896 patients died. Death occurred within 3 years in 1394 (13.8%) of patients. Death was adjudicated as cardiovascular in 737 patients, non-cardiovascular in 1166 patients, and undetermined in 993 patients. Associations with death or MI for indexed volumes and tissue density are shown in *Table 2*. Higher VAT density was associated with an increased risk of death or MI in both unadjusted [hazard ratio (HR) 1.40, 95% CI 1.37–1.43, *P* < .001] and adjusted (HR 1.24, 95% CI 1.19–1.28, *P* < .001) analyses. Higher SAT density, higher EAT density, and higher IMAT density were also associated with increased risk in multivariable analysis, with adjusted HR ranging from 1.10 to 1.25, and significantly improved model fit (all *P* < .001). Kaplan–Meier curves for low compared to high VAT density and low compared to high SM volume index are shown in *Figure 2*. Patients with high VAT density were at almost double the risk of death or MI (HR 1.85, 95% CI 1.73–1.99), and patients with low SM volume index were at 46% higher risk (HR 1.46, 95% CI 1.36–1.57).

**Table 2:** Associations with death or myocardial infarction for elevated values of tissue volume and density

	Unadjusted HR (95% CI)	*P*-value	Adjusted HR (95% CI)	*P*-value	Increase in LR χ^2^
SM volume	**0.88 (0.85–0.91)**	**<**.**001**	**0.91** (**0.87–0.95)**	**<**.**001**	**18**.7*
SM density	**0.94** (**0.92–0.95)**	**<**.**001**	**0.93** (**0.91–0.96)**	**<**.**001**	**15**.3*
Bone volume	**1.12** (**1.08–1.16)**	**<**.**001**	**0.88** (**0.83–0.92)**	**<**.**001**	**29**.7*
Bone density	**0.80** (**0.78–0.83)**	**<**.**001**	**0.88** (**0.84–0.91)**	**<**.**001**	**51**.1*
SAT volume	**0.84** (**0.81–0.88)**	**<**.**001**	**0.89** (**0.84–0.95)**	**<**.**001**	**14**.4*
SAT density	**1.27** (**1.23–1.31)**	**<**.**001**	**1.22** (**1.17–1.27)**	**<**.**001**	**93**.2*
EAT volume	**1.07** (**1.04–1.09)**	**<**.**001**	1.01 (0.97–1.05)	.580	0.30
EAT density	1.03 (0.99–1.07)	.093	**1.10** (**1.06–1.14)**	**<**.**001**	**22**.7*
VAT volume	**1.04** (**1.01–1.08)**	.**021**	**0.91** (**0.87–0.95)**	**<**.**001**	**17**.1*
VAT density	**1.40** (**1.37–1.43)**	**<**.**001**	**1.24** (**1.19–1.28)**	**<**.**001**	**126**.4*
IMAT volume	**1.04** (**1.01–1.08)**	.**010**	**0.95** (**0.91–0.99)**	.**020**	5.6
IMAT density	**1.29** (**1.24–1.35)**	**<**.**001**	**1.25** (**1.20–1.31)**	**<**.**001**	**101**.3*

Hazard ratios (HRs) reflect the risk per standard deviation increase in value. The multivariable model included age, sex, body mass index, medical history, coronary artery calcium, stress total perfusion deficit, stress left ventricular ejection fraction, change in ejection fraction, myocardial flow reserve, and revascularization within 90 days. Each body composition component was evaluated separately. Significant associations in bold.

CI, confidence interval; EAT, epicardial adipose tissue; IMAT, intermuscular adipose tissue; SAT, subcutaneous adipose tissue; SM, skeletal muscle; VAT, visceral adipose tissue.

^*^Indicates significant increase in likelihood ratio (LR) χ^2^ (*P* < .001 to account for multiple testing).

**Figure 3:**
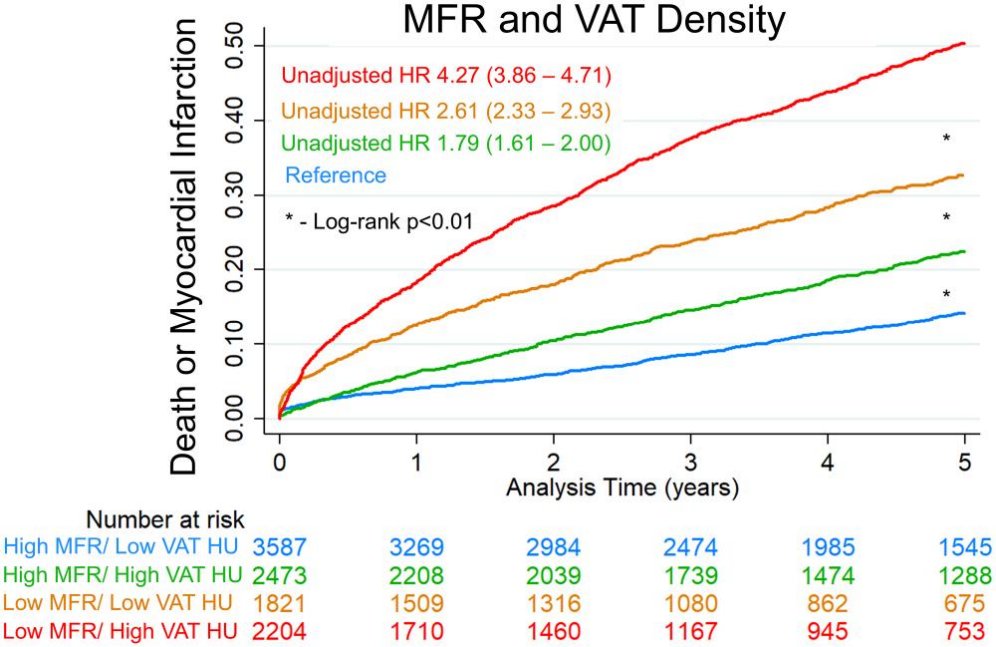
Unadjusted Kaplan–Meier curves as a function of myocardial flow reserve (MFR) and visceral adipose tissue (VAT) density. Abnormal MFR was defined as <2. Abnormal VAT density was based on sex-specific thresholds defined above. Hazard ratios (HRs) with 95% confidence intervals are shown. HU, Hounsfield units

The associated risk from abnormal body composition was incremental to the risk from other PET markers such as MFR, as shown in *Figure 3*. Compared to patients with preserved MFR and low VAT density, patients with both elevated VAT density and reduced MFR were at higher risk of death or MI (unadjusted HR 4.27, 95% CI 3.86–4.71). After adjusting for the other components of the multivariable model, patients with both elevated VAT density and reduced MFR continued to be at higher risk of death or MI (adjusted HR 2.49, 95% CI 2.23–2.77, *P* < .001). Patients with elevated VAT density and preserved MFR (adjusted HR 1.57, 95% CI 1.40–1.75, *P* < .001) or with low VAT density and reduced MFR (adjusted HR 1.87, 95% CI 1.67–2.11, *P* < .001) were also at increased risk. Associations assessed separately in female and male patients are shown in Supplementary data online, *Table S6*. Associations with adipose tissue density were similar in both sexes, but SM volume index was only independently associated with death or MI in male patients. The patterns of associations were generally similar in patients above or below age 65, as shown in Supplementary data online, *Table S7*. However, there were some differences. For example, higher bone and SAT volumes were associated with decreased risk in patients age ≥ 65, but not in patients under 65 (although interaction terms were not significant; *P* = .081 and *P* = .557, respectively). Higher EAT density was associated with increased risk in patients age ≥ 65, but not in patients under 65 (interaction *P* = .243). Associations with MI, death, and cardiovascular death (as separate outcomes) are shown in Supplementary data online, *Table S8*. Associations with death or MI for volumes indexed by BSA compared to volumes expressed as percentage of the total thoracic volume are shown in Supplementary data online, *Table S9*. This analysis demonstrated similar associations with each method in unadjusted analyses, but better model fit for volumes indexed to BSA in adjusted analyses. Total thoracic volume (adjusted HR 0.87, 95% CI 0.81–0.91, *P* < .001) and lung volumes (adjusted HR 0.88, 95% CI 0.85–0.92, *P* < .001) are independently associated with death or MI, while BSA is not (adjusted HR 1.00, 95% CI 0.94–1.07, *P* = .903).

**Figure 4:**
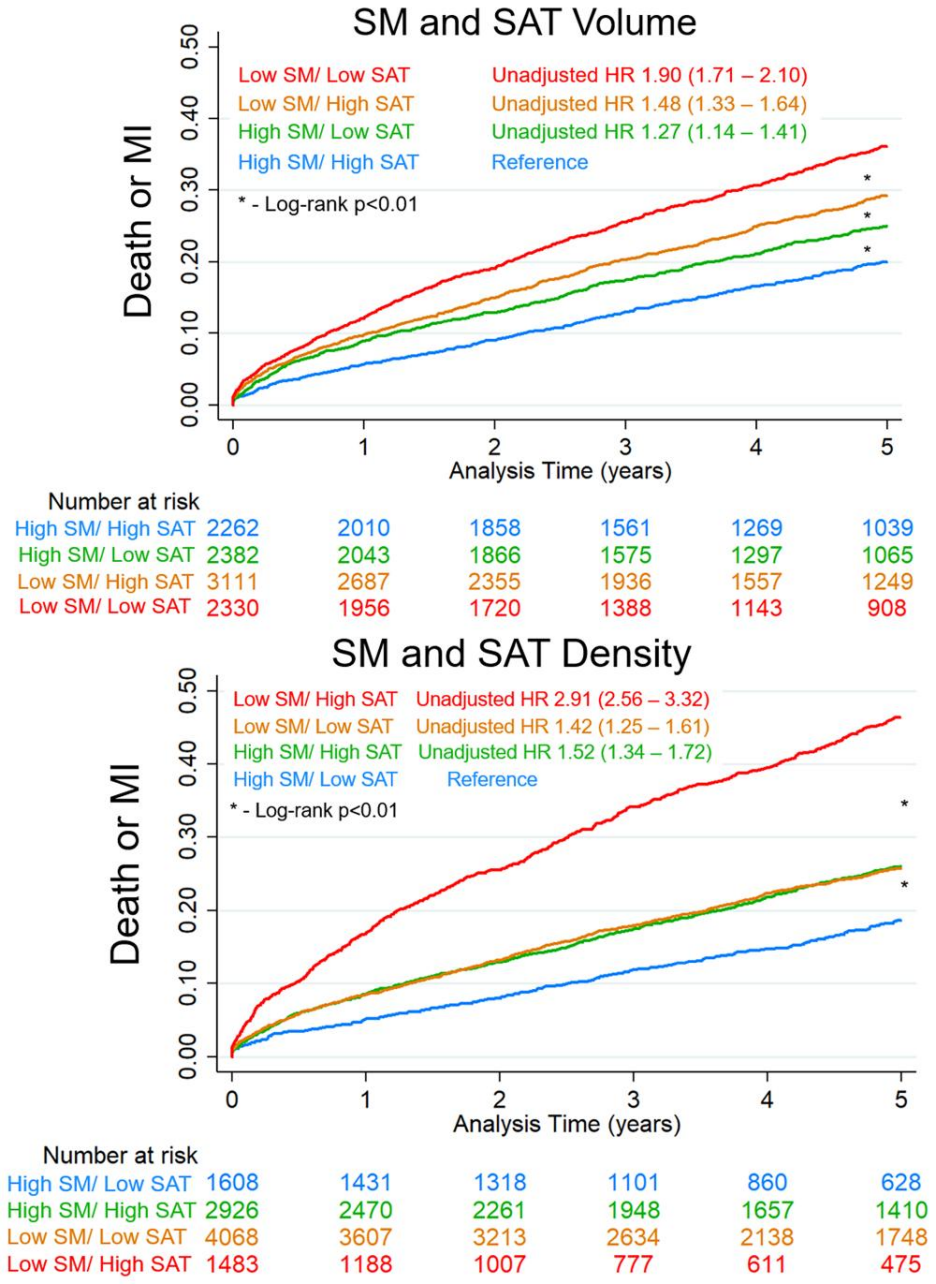
Unadjusted Kaplan–Meier survival curves for death or myocardial infarction (MI). Patients were classified according to skeletal muscle (SM) and subcutaneous adipose tissue (SAT) volume and density. Hazard ratios (HRs) with 95% confidence intervals are shown

There was also incremental benefit from considering more than one tissue component as outlined in *Figure 4*. Patients with low SM volume index and low SAT volume index had a significantly higher risk of death or MI (unadjusted HR 1.90, 95% CI 1.71–2.10, *P* < .001), with less risk associated with low values for SM or SAT volume index in isolation. Similarly, the risk associated with abnormal SM (low) and SAT (high) density (unadjusted HR 2.91, 95% CI 2.56–3.32) was significantly higher than abnormal SM or SAT density in isolation.

**Figure 5:**
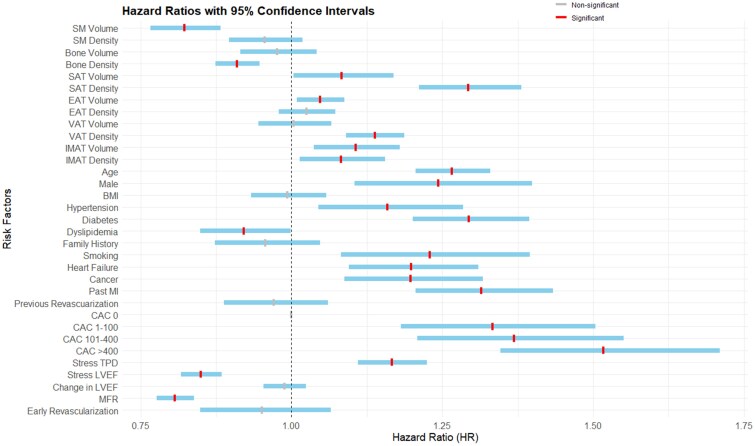
Adjusted associations with death or myocardial infarction (MI) in the multivariable model incorporating segmentations as continuous variables. Adjusted hazard ratios shown in red if significant and grey if non-significant. The hazard ratios for continuous variables are expressed as per standard deviation increase in value. Confidence intervals in blue. BMI, body mass index; CAC, coronary artery calcium; EAT, epicardial adipose tissue; IMAT, intermuscular adipose tissue; LVEF, left ventricular ejection fraction; MFR, myocardial flow reserve; SAT, subcutaneous adipose tissue; SM, skeletal muscle; TPD, total perfusion deficit; VAT, visceral adipose tissue

Associations with death or MI for body tissue components modelled as continuous variables are shown in *Figure 5*, with additional details shown in Supplementary data online, *Table S10*. Of note, BMI was not associated with death or MI in the multivariable model (adjusted HR 0.99, 95% CI 0.93–1.06, *P* = .838).

## Discussion

In this large, multicentre study, we explored the correlations and potential clinical utility of volumetric body composition measurements. We demonstrated that deep learning can rapidly quantify multiple body tissue components (SM, bone, and four adipose tissues) from ancillary CT attenuation imaging performed during PET scan. Lower values for SM indexed volume were associated with risk of death or MI. However, we also identified that abnormalities in the density of all adipose tissues and SM were associated with death or MI. Importantly, these associations persisted after adjusting for extent of coronary atherosclerosis (CAC), functional significance of CAD (MFR), age, sex, and clinical risk factors, including BMI, suggesting that quantitative body composition provides critical information on cardiometabolic health separate from traditional risk measures (*Structured Graphical Abstract*). This new information can be obtained rapidly and automatically from existing scans without extra imaging time or radiation, allowing physicians to identify patients at increased cardiovascular risk.

Our analysis included assessment of correlations with other variables to determine if our automatic analysis of thoracic body composition can provide clinicians with novel clinical insights. The majority of correlations with age, BMI, TPD, CAC, and MFR was weak, indicating that volumetric body composition measures offer new insights. Notably, only SAT-indexed volume and density showed modest correlations with BMI. Interestingly, we found contrasting associations between SAT with CAC (weak negative correlation) compared to EAT and VAT (weak positive correlations). This discrepancy likely reflects the link between ectopic adipose tissue deposition and cardiometabolic risk factors.^32^ Additionally, we noted a weak positive correlation between SM volume with MFR and a weak negative correlation between VAT density and MFR. Souza *et al*.^19^ recently demonstrated in a smaller single centre sample (*n* = 400 with 86 events over 6 years—representing <4% of our multicentre population), that SM volume from separate abdominal CT obtained on abdominal pain indications was associated with abnormal MFR, but did not identify an association between SAT or VAT. This discrepancy may be related to the study being underpowered for such analysis, or due to utilizing abdominal CT instead of volumetric chest CT data, with thoracic VAT measurements being more closely related to the local adverse paracrine effects.^33^ Alternatively, this could be related to relying on measurements from a single axial slice compared to volumetric measurements (as was done in the present study) and selection bias due to difference indications. There were modest correlations between tissue volume index and density with age. The relationships between body composition measures and death or MI were generally similar by age, with some differences between age categories. Volumes indexed to BSA demonstrated significant associations with death or MI more consistently and had better improvement in model fit compared to volumes expressed as a percentage of the thoracic volume in adjusted analyses. This may be a reflection of the independent prognostic importance of total thoracic volume (which is driven primarily by lung volumes). Regardless, our findings in combination with previous studies suggest that body tissue composition measurements provide objective measures of sarcopenia and cardiometabolic health.

We evaluated the associations between body tissue composition and death or MI to evaluate their potential clinical utility. Using sex-specific thresholds for normal values, low SM volume index was associated with risk of death or MI. This finding was observed in both female and male patients. However, SM volume index was only independently associated with death or MI in male patients. We found that age and history of heart failure or cancer were associated with low SM volume index. Therefore, SM volume index may reflect the biologic age while also measuring the systemic effects of chronic disease. Sarcopenia, defined as an age-related loss of SM mass or function, and cachexia (low SM and adipose tissue volume) are associated with physical disability^34,35^ and with mortality in patients with heart failure and other cardiovascular disorders.^35,36^ However, objective quantification is rarely performed and typically relies on area measurements of a limited number of tissues from a single slice of a CT scan.^19,37–40^ For example, Souza *et al*.^19^ evaluated area measurements of SM, SAT, and VAT on abdominal CT slice at the L3 level. Nachit *et al*.^39^ evaluated area measurements of SM at the L3 level and SAT, and VAT at the L1 level on abdominal CT. Xu *et al*.^38^ evaluated SM and SAT area at the T5, T8, and T10 levels from lung cancer screening chest CT. Other groups have evaluated SM area measured on CT at the mid-thigh level.^41^ In contrast, we provide opportunistic measures obtained automatically from the cardiac CT attenuation maps, from patients undergoing cardiac PET/CT without the need for an additional abdominal scan. Additionally, we perform volumetric measurements (with automated standardization of *z*-axis coverage) to provide a more complete characterization of tissue volumes. Similar volume measurements could be obtained from magnetic resonance imaging,^42^ but this would not capture the density information also captured by CT. In unadjusted analysis, having lower SM density was also associated with an increased risk of death or MI. Higher SM density is associated with greater force production,^43^ and therefore can be considered a measure of muscle quality which also has implications for sarcopenia and frailty. Additionally, previous analyses have shown that myosteatosis (in patients undergoing abdominal CT for colon screening) is associated with mortality.^39^ The thresholds for myosteatosis (<19 HU for female patients and <28 HU for male patients) in that single centre analysis of abdominal CT are very similar to the thresholds for abnormal SM density identified in this multicentre analysis using CTAC scans of the chest. Importantly, patients with sarcopenia may benefit from targeted interventions such as cardiac rehabilitation.^44^ Therefore, physicians may be able to leverage this information to target interventions to the highest risk patients.

Associations with adiposity are more complex; however, they are important to consider given the rapidly expanding global epidemic of obesity^45^ and steeply rising rates of metabolic syndrome.^46^ Body mass index is frequently utilized to evaluate adiposity but has only modest correlations with adipose tissue volumes in many studies including this work. Importantly, our results suggest that not all adipose tissue carries similar risk. We found that patients with higher SAT volumes had a lower risk of death or MI, while higher volumes of other adipose tissues were associated with an increased risk. In fact, high SAT volume was independently associated with lower risk of the composite outcome as well as the individual components in multivariable analyses. These differences were expected, given the known differences in cardiometabolic risk for ectopic adipose deposition.^33^ Future studies using volumetric body composition could potentially provide important insights into the apparent obesity paradox by comparing distributions of adipose tissue.^47^

In addition to indexed volumes, we evaluated associations with adipose tissue density. Importantly, the subtle differences in adipose tissue density would not be readily apparent during routine clinical interpretation of the images and therefore this information would not be available without an approach such as the one presented here. We found that heart failure and diabetes were associated with elevated VAT density, suggesting that it may be a global measure of cardiometabolic health. Adipose tissue density was consistently associated with risk of death or MI, even after correcting for age, sex, medical history, and imaging findings (including CAC and MFR). These findings were consistent in female and male patients as well as for both all-cause mortality and cardiovascular mortality. They are also consistent with our preliminary body composition analyses in patients undergoing single photon emission computed tomography.^48^ Importantly, since MFR measures are obtained during PET imaging, we are able to account for microvascular dysfunction, which is common in patients with cardiometabolic disease.^49^ Further, the associations with adipose tissue density were also independent of age, medical history (including heart failure and cancer), and BMI demonstrating the robust, independent risk stratification which can be achieved by considering body composition. Lastly, we demonstrate that the associations with adipose tissue density were also present with cardiovascular death. This is a critical finding for clarifying the potential clinical utility of body composition analysis in a PET MPI population which has a high proportion of older high-risk patients, as well as a higher proportion of cardiometabolic disease—patient groups where risk stratification is particularly crucial. Higher EAT density may be a marker of coronary inflammation^50^ and is correlated with sodium fluoride uptake,^51^ which is a measure of microcalcification that reflects atherosclerotic plaque activity.^52^ Indeed, EAT interacts with the heart through local paracrine effects and inflammatory signalling.^53^ Similarly, VAT density could be elevated in the presence of inflammation.^54^ Therefore, adipose tissue density may provide insights into the overall inflammatory state. Importantly, we derived sex-specific thresholds for abnormal values for indexed volume or density which could be incorporated into deep learning-enabled body composition reports. These values are similar to thresholds established in our recent analysis of single-photon emission computed tomography (SPECT) MPI patients.^48^ This approach could allow physicians to consider targeted interventions such as cardiac rehabilitation for patients with low SM volume or density or more aggressive cardiometabolic risk factor management in patients with abnormal adipose tissue density.

Our study has a few important limitations. Given the large number of patients, we did not perform an evaluation of manual segmentation for comparison. However, it is not practical to manually segment these volumes in clinical practice and therefore the comparison would not provide insights into differences in clinical performance. Secondly, we do not have other clinical measures of sarcopenia to compare to the quantitative measures that we derived. Clinical applications of this approach would benefit from age and sex-specific normal population distributions in healthy populations. However, to date, CT body composition quantification (including the present study) has been evaluated in patients undergoing CT imaging for clinical indications. Our analysis, which reflected this consideration, focused on associated risk as a function of continuous values to explore the clinical value of body composition quantification. We dichotomized the population to provide further clinical context, similar to previous studies,^19^ while other groups have split populations according to interquartile range.^38^ Regardless of the approach, these cut-offs may not be applicable to other patient populations. Ultimately large studies in healthy populations are needed to establish expected distributions of body composition measures so that this information can be more generalizable. While the risk of death in our population was similar to other PET referral populations,^4^ further evaluation in lower risk populations is needed. Xu *et al*.^38^ recently demonstrated that area-based measurements of body composition could improve prediction of cardiovascular and all-cause mortality in a cohort of patients undergoing lung cancer screening chest CT. Fat to muscle ratio (from bioimpedance measurements) was associated with all-cause mortality in the United Kingdom BioBank dataset.^55^ These results suggest that our findings likely extend to other imaging populations. We utilized deep learning CAC from CT attenuation imaging. While these measurements have good concordance with expert CAC annotations from dedicated electrocardiogram-gated scans,^15^ they are derived from low-dose ungated scans which may limit the accuracy of the CAC measurements. Lastly, given the observational nature of the study, residual confounding is possible even though we adjusted for multiple clinically important risk factors.

Volumetric body tissue composition can be rapidly and automatically evaluated using deep learning on existing low-dose CT attenuation scans. These data provide detailed, quantitative assessments of sarcopenia and insights into metabolic health, which offer independent and incremental risk stratification compared to traditional measures such as age, BMI, and PET markers.

## Supplementary Material

ehaf131_Supplementary_Data

## Declarations

### Data Availability

To the extent allowed by data sharing agreements and institutional review board protocols, the data from this manuscript will be shared upon written request.

### Pre-registered Clinical Trial Number

None supplied.
