# Supplementary material for: Deep learning-quantified body composition from positron emission tomography/computed tomography and cardiovascular outcomes: a multicentre study
Source: Eur Heart J. 2025 Mar 30;46(24):2336–47. doi: 10.1093/eurheartj/ehaf131 (PMC12190801; doi:10.1093/eurheartj/ehaf131)
Supplement: ehaf131_Supplementary_Data [file ehaf131_supplementary_data.docx]

**SUPPLEMENTARY MATERIAL**

**
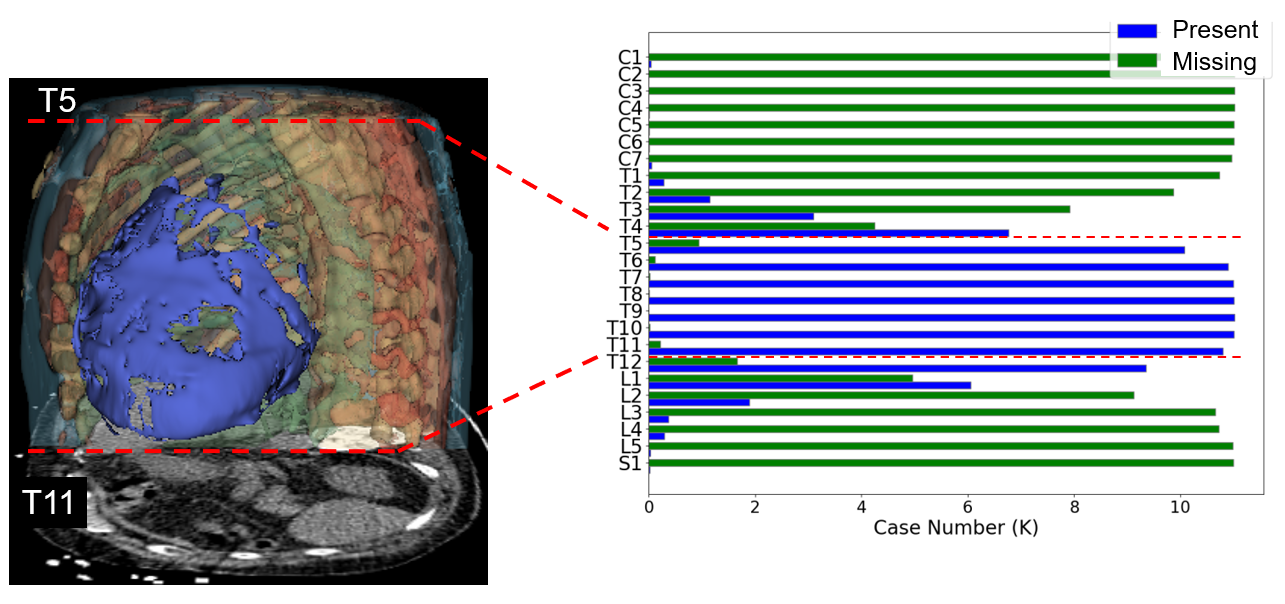
**

**Supplementary Figure 1:** Proportion of missing data as a function of vertebral height. The vertebra information was automatically obtained from the deep learning segmentation^26^. Over 90% of cases had available data encompassing the T5-T11 vertebrae, which was used as the limit for body composition segmentation.


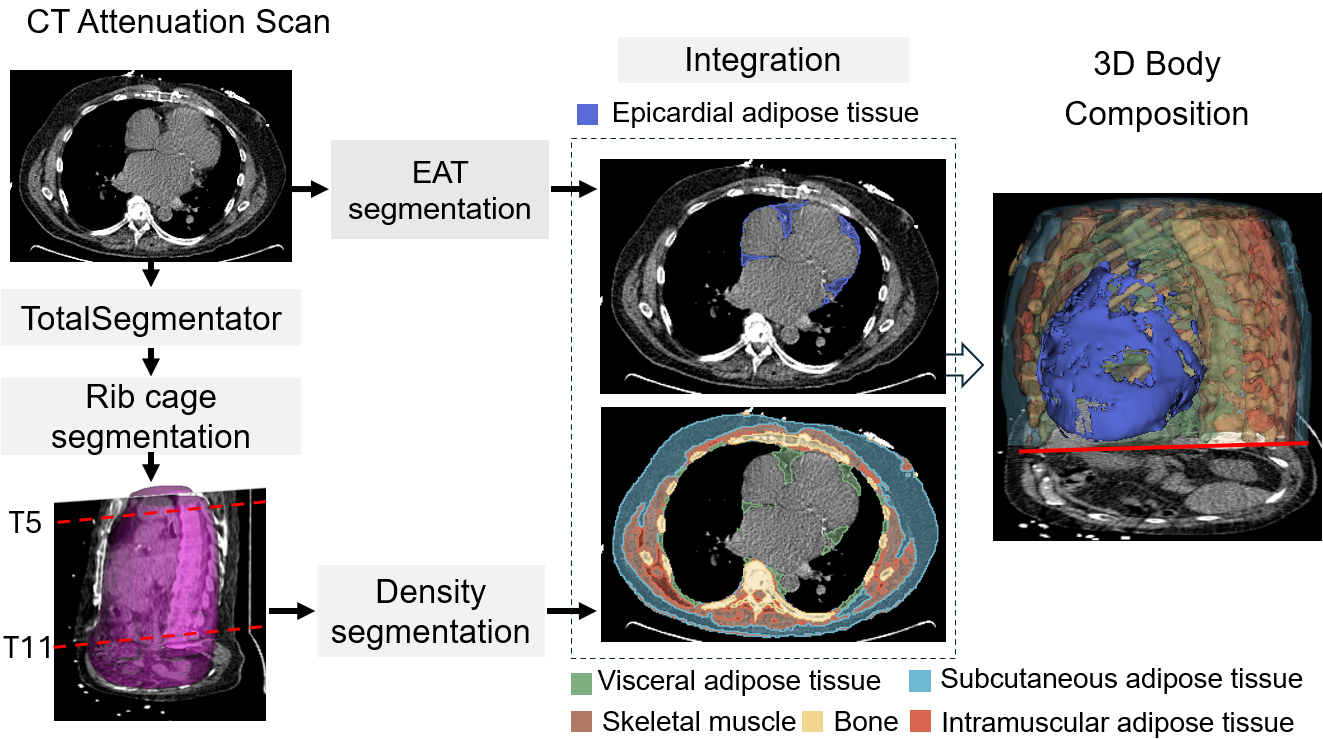


**Supplementary Figure 2:** Overview of 3D body composition segmentation. T5-T11 volume is automatically determined from the segmented vertebrae by a previously trained model (TotalSegmentator). TotalSegmentator was also used to define chest organs. Image processing combining multiple segmented organs was applied to define the thoracic cavity. Subsequently, tissue components are defined based on CT density and their relationship to the thoracic cavity. Epicardial adipose tissue (EAT) was segmented using a previously validated convolutional long short-term memory model^15^.


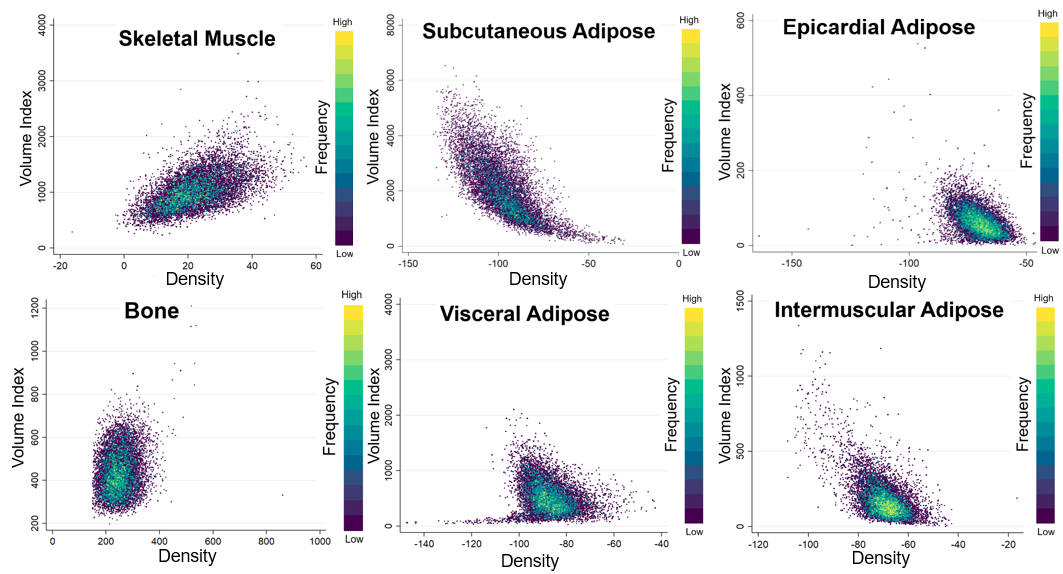


**Supplementary Figure 3:** Distribution of body composition volume index and density.

**
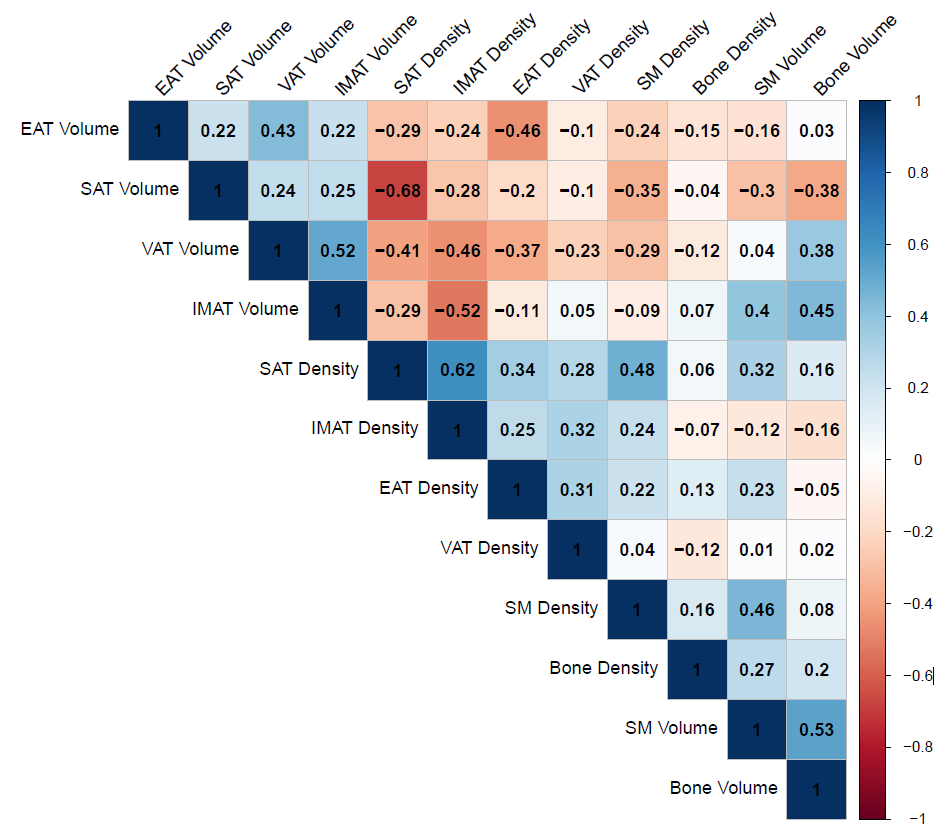
**

**Supplementary Figure 4:** Correlation between tissue volumes indexed to body surface area and density. Shades of blue indicate a positive correlation, and red indicates a negative correlation. Increasing darkness depicts an increasing strength of correlation. Features were ordered using hierarchical clustering. EAT – epicardial adipose tissue, IMAT – intermuscular adipose tissue, SAT – subcutaneous adipose tissue, SM – skeletal muscle, VAT – visceral adipose tissue.


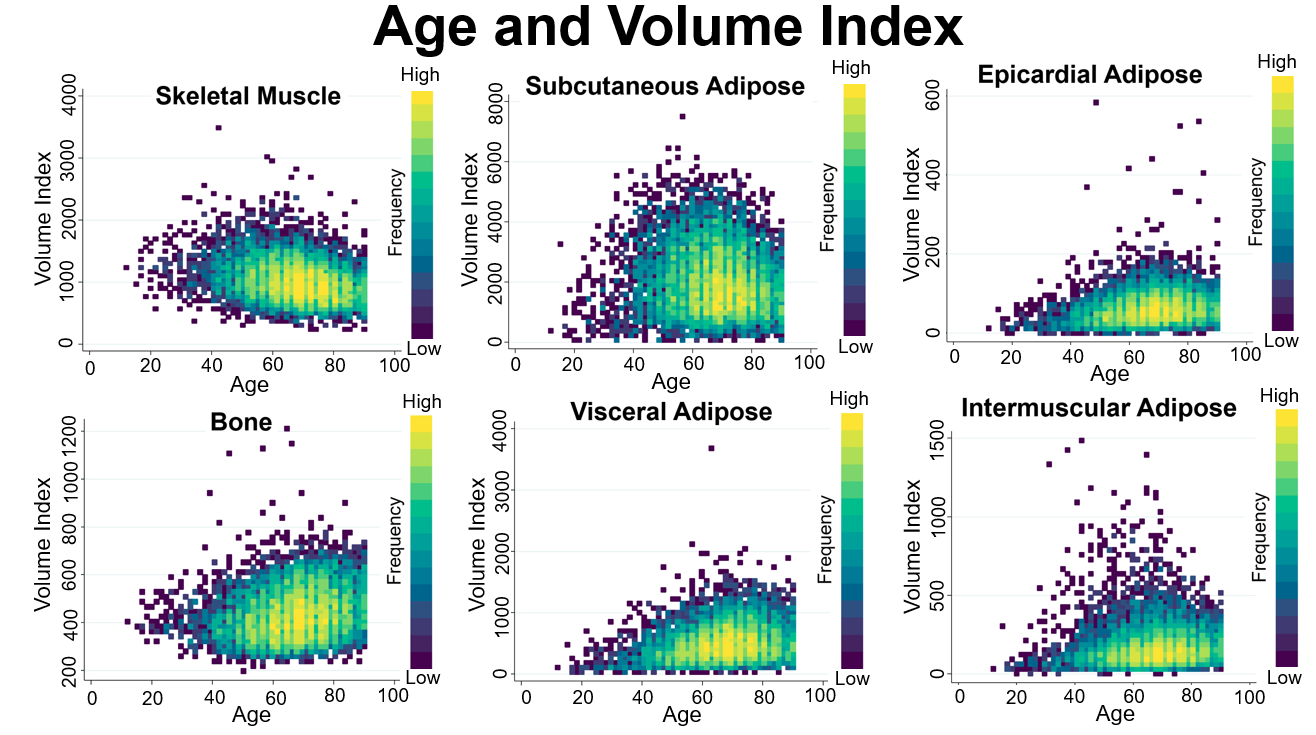


**Supplementary Figure 5:** Distribution of body composition volume index and age


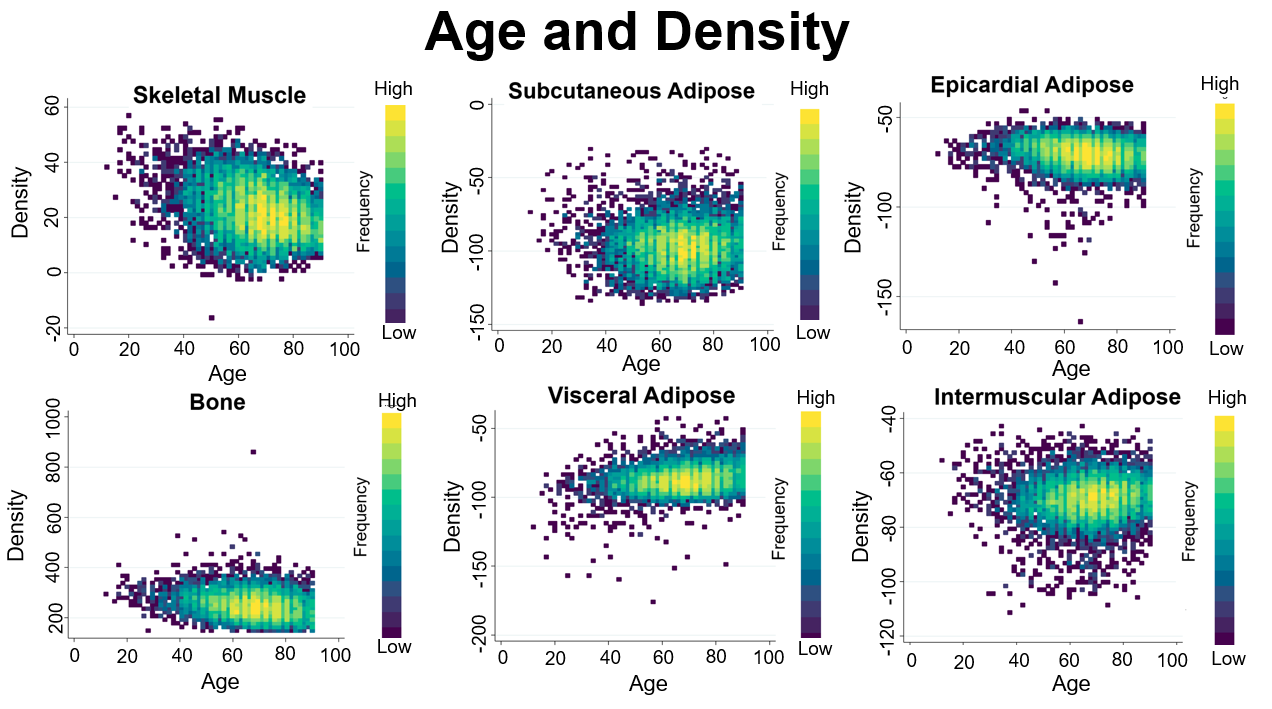


**Supplementary Figure 6:** Distribution of body composition density and age


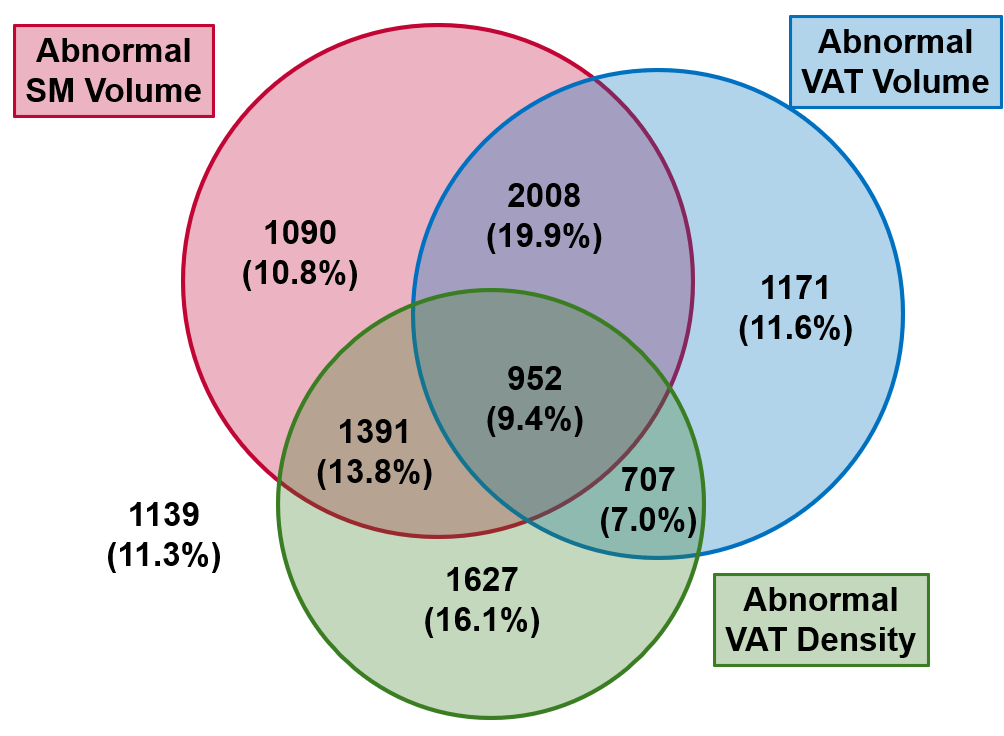


**Supplementary Figure 7:** Venn diagram outlining relationships between abnormal body composition. SM – skeletal muscle, VAT – visceral adipose tissue.

Supplementary Table 1

| Site | kVp | Tube current (mAs) | Spacing x (mm) | Spacing y (mm) | Spacing z (mm) | Dimensionality x | Dimensionality y | Dimensionality z |
| --- | --- | --- | --- | --- | --- | --- | --- | --- |
| Mayo | 120-140 | 20-180 | 1.37 | 1.37 | 3.27 | 512 | 512 | 27-47 |
| BWH | 80-140 | 10-300 | 0.98-1.37 | 0.98-1.37 | 2.5-5 | 512 | 512 | 47 |
| CSMC | 100 | 11-13 | 1.37 | 1.37 | 3 | 512 | 512 | 39-75 |

**Supplementary Table 1:** Imaging parameters at three sites. BWH – Brigham and Women’s Hospital, CSMC – Cedars-Sinai Medical Center

Supplementary Table 2

|  | Age | BMI | Stress TPD | CAC | MFR |
| --- | --- | --- | --- | --- | --- |
| SM Volume index | **-0.288** | **-0.060** | **0.042** | 0.004 | **0.072** |
| SM Density | **-0.256** | **-0.251** | **0.047** | **-0.078** | **0.060** |
| Bone Volume index | **0.214** | **-0.310** | **0.078** | **0.229** | 0.018 |
| Bone Density | **-0.238** | **0.039** | -0.027 | **-0.040** | **0.042** |
| SAT Volume index | **-0.082** | **0.684** | **-0.096** | **-0.174** | -0.008 |
| SAT Density | -0.009 | **-0.439** | **0.080** | **0.098** | -0.017 |
| EAT Volume index | **0.152** | **0.231** | **0.063** | **0.081** | -0.033 |
| EAT Density | **-0.169** | **-0.116** | -0.012 | **-0.102** | 0.010 |
| VAT Volume index | **0.222** | **0.250** | **0.030** | **0.116** | 0.001 |
| VAT Density | **0.211** | **-0.110** | **0.083** | **0.092** | **-0.064** |
| IMAT Volume index | **0.046** | **0.276** | 0.017 | 0.027 | 0.005 |
| IMAT Density | **0.046** | **-0.258** | 0.022 | 0.018 | -0.030 |

**Supplementary Table 2:** Correlation between body tissue composition and selected clinical and imaging features. Significant correlations (p<0.0001 to account for multiple testing) shown in bold. BMI – body mass index, CAC – coronary artery calcium, EAT – epicardial adipose tissue, IMAT – intermuscular adipose tissue, MFR – myocardial flow reserve, SAT – subcutaneous adipose tissue, SM – skeletal muscle, TPD – total perfusion deficit, VAT – visceral adipose tissue.

Supplementary Table 3

|  | Adjusted OR | p-value |
| --- | --- | --- |
| **Age (per SD increase)** | **1.89 (1.80 – 1.99)** | **<0.001** |
| **Male** | **0.79 (0.72 - 0.86)** | **<0.001** |
| **Body mass index (per SD increase)** | **1.25 (1.19 – 1.31)** | **<0.001** |
| Hypertension | 0.97 (0.86 - 1.08) | 0.557 |
| Diabetes | 0.92 (0.84 – 1.00) | 0.062 |
| Dyslipidemia | 1.01 (0.91 - 1.11) | 0.890 |
| **Family History** | **1.13 (1.02 - 1.26)** | **0.021** |
| **Smoking** | **1.34 (1.15 - 1.57)** | **<0.001** |
| **Heart Failure** | **1.21 (1.08 - 1.37)** | **0.001** |
| **Cancer** | **1.33 (1.19 - 1.49)** | **<0.001** |
| Past myocardial infarction | 0.92 (0.81 - 1.03) | 0.147 |
| **Previous revascularization** | **0.68 (0.61 - 0.77)** | **<0.001** |
| CAC 0 | Reference | - |
| CAC 1-100 | 0.96 (0.84 - 1.09) | 0.530 |
| **CAC 101-400** | **1.19 (1.04 - 1.37)** | **0.014** |
| **CAC > 400** | **1.37 (1.20 - 1.56)** | **<0.001** |
| **Log stress total perfusion deficit + 1 (per SD increase)** | **1.10 (1.04 - 1.17)** | **0.001** |
| **Log Stress LVEF (per SD increase)** | **0.95 (0.91 - 0.99)** | **0.028** |
| **Change in LVEF (per SD increase)** | **1.08 (1.02 - 1.14)** | **0.009** |
| **Log Myocardial flow reserve (per SD increase)** | **0.83 (0.79 - 0.87)** | **<0.001** |
| **Early revascularization** | **0.83 (0.71 - 0.96)** | **0.014** |

**Supplementary Table 3**: Associations with low skeletal muscle volume index. Significant associations in bold. CAC – coronary artery calcium, LVEF – left ventricular ejection fraction, OR – odds ratio, SD – standard deviation.

Supplementary Table 4

|  | Adjusted OR | p-value |
| --- | --- | --- |
| **Age (per SD increase)** | **1.22 (1.16 – 1.28)** | **<0.001** |
| **Male** | **0.79 (0.72 - 0.86)** | **<0.001** |
| **Body mass index (per SD increase)** | **0.76 (0.73 – 0.80)** | **<0.001** |
| **Hypertension** | **1.27 (1.13 - 1.42)** | **<0.001** |
| **Diabetes** | **1.12 (1.02 - 1.23)** | **0.016** |
| **Dyslipidemia** | **0.73 (0.66 - 0.81)** | **<0.001** |
| **Family History** | **0.82 (0.74 - 0.91)** | **<0.001** |
| Smoking | 1.00 (0.86 - 1.17) | 0.994 |
| **Heart Failure** | **1.34 (1.19 - 1.50)** | **<0.001** |
| **Cancer** | **0.72 (0.65 - 0.81)** | **<0.001** |
| Past myocardial infarction | 1.10 (0.98 - 1.23) | 0.119 |
| Previous revascularization | 1.03 (0.92 - 1.16) | 0.573 |
| CAC 0 | Reference | - |
| CAC 1-100 | 1.02 (0.90 - 1.16) | 0.728 |
| CAC 101-400 | 0.91 (0.79 - 1.04) | 0.172 |
| CAC > 400 | 0.95 (0.84 - 1.09) | 0.473 |
| **Log stress total perfusion deficit + 1 (per SD increase)** | **0.86 (0.81 - 0.91)** | **<0.001** |
| **Log Stress LVEF (per SD increase)** | **1.11 (1.07 - 1.17)** | **<0.001** |
| **Change in LVEF (per SD increase)** | **0.68 (0.64 - 0.72)** | **<0.001** |
| **Log Myocardial flow reserve (per SD increase)** | **0.78 (0.74 - 0.82)** | **<0.001** |
| **Early revascularization** | **0.72 (0.61 - 0.83)** | **<0.001** |

**Supplementary Table 4:** Associations with elevated visceral adipose tissue density. Significant associations in bold. CAC – coronary artery calcium, LVEF – left ventricular ejection fraction, OR – odds ratio, SD – standard deviation.

Supplementary Table 5

|  | Adjusted OR | p-value |
| --- | --- | --- |
| **SM Volume index (per SD increase)** | **0.74 (0.68 - 0.81)** | **<0.001** |
| **SM Density (per SD increase)** | **0.88 (0.81 - 0.96)** | **0.003** |
| Bone Volume index (per SD increase) | 0.99 (0.91 - 1.08) | 0.852 |
| **Bone Density (per SD increase)** | **0.92 (0.87 - 0.97)** | **0.001** |
| SAT Volume index (per SD increase) | 1.03 (0.93 - 1.14) | 0.571 |
| **SAT Density (per SD increase)** | **1.38 (1.26 - 1.51)** | **<0.001** |
| EAT Volume index (per SD increase) | 1.01 (0.96 - 1.07) | 0.584 |
| EAT Density (per SD increase) | 1.02 (0.97 - 1.08) | 0.476 |
| VAT Volume index (per SD increase) | 1.01 (0.93 - 1.08) | 0.891 |
| **VAT Density (per SD increase)** | **1.30 (1.23 - 1.38)** | **<0.001** |
| **IMAT Volume index (per SD increase)** | **1.13 (1.05 - 1.23)** | **0.002** |
| IMAT Density (per SD increase) | 0.95 (0.88 - 1.04) | 0.275 |
| **Age (per SD increase)** | **1.14 (1.07 - 1.21)** | **<0.001** |
| Male | 0.90 (0.77 - 1.05) | 0.188 |
| Body mass index (per SD increase) | 0.95 (0.88 - 1.03) | 0.190 |
| Hypertension | 1.21 (1.07 - 1.37) | 0.003 |
| **Diabetes** | **1.77 (1.60 - 1.95)** | **<0.001** |
| **Dyslipidemia** | **0.89 (0.80 - 0.99)** | **0.031** |
| **Family History** | **0.85 (0.76 - 0.96)** | **0.007** |
| **Smoking** | **0.66 (0.55 - 0.78)** | **<0.001** |
| **Heart Failure** | **1.27 (1.12 - 1.43)** | **<0.001** |
| Cancer | 0.91 (0.80 - 1.03) | 0.120 |
| Past myocardial infarction | 1.06 (0.93 - 1.20) | 0.395 |
| **Previous revascularization** | **0.82 (0.72 - 0.93)** | **0.002** |
| CAC 0 | Reference | - |
| CAC 1-100 | 1.03 (0.89 - 1.19) | 0.658 |
| CAC 101-400 | 1.03 (0.89 - 1.20) | 0.654 |
| **CAC > 400** | **1.36 (1.18 - 1.57)** | **<0.001** |
| **Log stress total perfusion deficit + 1 (per SD increase)** | **1.61 (1.51 - 1.72)** | **<0.001** |
| **Log Stress LVEF (per SD increase)** | **0.74 (0.70 - 0.78)** | **<0.001** |
| Change in LVEF (per SD increase) | 0.95 (0.90 - 1.01) | 0.138 |
| **Early revascularization** | **1.77 (1.51 - 2.09)** | **<0.001** |

**Supplementary Table 5:** Associations with reduced myocardial flow reserve (<2). Significant associations in bold. CAC – coronary artery calcium, EAT – epicardial adipose tissue, IMAT – intramuscular adipose tissue, LVEF – left ventricular ejection fraction, OR – odds ratio, SAT – subcutaneous adipose tissue, SD – standard deviation, SM – skeletal muscle, VAT – visceral adipose tissue.

Supplementary Table 6

| Unadjusted analyses | | | | |
| --- | --- | --- | --- | --- |
|  | Female patients | | Male Patients | |
|  | Unadjusted HR (95% CI) | p-value | Unadjusted HR (95% CI) | p-value |
| SM Volume per SD increase | **0.92 (0.85 – 0.99)** | **0.023** | **0.71 (0.38 – 0.75)** | **<0.001** |
| SM Density per SD increase | **0.71 (0.66 – 0.76)** | **<0.001** | **0.94 (0.93 – 0.96)** | **<0.001** |
| Bone Volume per SD increase | **1.14 (1.06 – 1.22)** | **<0.001** | 0.99 (0.94 – 1.04) | 0.698 |
| Bone Density per SD increase | **0.78 (0.73 – 0.82)** | **<0.001** | **0.82 (0.78 – 0.86)** | **<0.001** |
| SAT Volume per SD increase | **0.87 (0.82 – 0.92)** | **<0.001** | **0.91 (0.86 – 0.96)** | **0.001** |
| SAT Density per SD increase | **1.33 (1.26 – 1.41)** | **<0.001** | **1.21 (1.16 – 1.26)** | **<0.001** |
| EAT Volume per SD increase | **1.05 (1.02 – 1.09)** | **0.005** | **1.08 (1.04 – 1.12)** | **<0.001** |
| EAT Density per SD increase | 1.00 (0.95 – 1.06) | 0.865 | **1.08 (1.03 – 1.14)** | **0.003** |
| VAT Volume per SD increase | **1.11 (1.03 – 1.19)** | **0.004** | 0.94 (0.90 – 0.98) | 0.003 |
| VAT Density per SD increase | **1.56 (1.47 – 1.66)** | **<0.001** | **1.37 (1.33 – 1.41)** | **<0.001** |
| IMAT Volume per SD increase | **1.11 (1.04 – 1.19)** | **0.003** | 0.97 (0.93 – 1.01) | 0.146 |
| IMAT Density per SD increase | **1.71 (1.53 – 1.91)** | **<0.001** | **1.30 (1.24 – 1.36)** | **<0.001** |
| Multivariable analyses | | | | |
|  | Female patients | | Male Patients | |
|  | Adjusted HR (95% CI) | p-value | Adjusted HR (95% CI) | p-value |
| SM Volume per SD increase | 1.01 (0.94 – 1.08) | 0.842 | **0.85 (0.80 – 0.89)** | **<0.001** |
| SM Density per SD increase | **0.84 (0.77 – 0.92)** | **<0.001** | **0.95 (0.92 – 0.98)** | **0.001** |
| Bone Volume per SD increase | 0.96 (0.88 – 1.04) | 0.316 | **0.84 (0.79 – 0.89)** | **<0.001** |
| Bone Density per SD increase | **0.89 (0.84 – 0.94)** | **<0.001** | **0.87 (0.82 – 0.91)** | **<0.001** |
| SAT Volume per SD increase | **0.86 (0.80 – 0.94)** | **<0.001** | 0.93 (0.85 – 1.01) | 0.098 |
| SAT Density per SD increase | **1.25 (1.17 – 1.33)** | **<0.001** | **1.19 (1.13 – 1.25)** | **<0.001** |
| EAT Volume per SD increase | 1.02 (0.97 – 1.06) | 0.536 | 1.01 (0.96 – 1.07) | 0.645 |
| EAT Density per SD increase | **1.05 (0.99 – 1.11)** | **0.102** | **1.13 (1.08 – 1.20)** | **<0.001** |
| VAT Volume per SD increase | 1.00 (0.92 – 1.10) | 0.926 | **0.89 (0.84 – 0.93)** | **<0.001** |
| VAT Density per SD increase | **1.14 (1.07 – 1.22)** | **<0.001** | **1.27 (1.22 – 1.33)** | **<0.001** |
| IMAT Volume per SD increase | 1.00 (0.92 – 1.09) | 0.964 | **0.93 (0.89 – 0.98)** | **0.007** |
| IMAT Density per SD increase | **1.27 (1.18 – 1.38)** | **<0.001** | **1.24 (1.18 – 1.31)** | **<0.001** |

**Supplementary Table 6:** Associations with death or myocardial infarction stratified by patient sex. Hazard ratios (HR) reflect the risk per standard deviation (SD) increase. The multivariable model included age, sex, body mass index, medical history, coronary artery calcium, stress total perfusion deficit, stress left ventricular ejection fraction, change in ejection fraction, myocardial flow reserve, and revascularization within 90 days with each body composition component assessed separately. Significant associations in bold. CI – confidence interval, EAT – epicardial adipose tissue, IMAT – intermuscular adipose tissue, SAT – subcutaneous adipose tissue, SM – skeletal muscle, VAT – visceral adipose tissue.

Supplementary Table 7

|  | Patients < 65 years N = 4093) | | Patients ≥ 65 years (N = 5992) | |
| --- | --- | --- | --- | --- |
|  | Unadjusted HR (95% CI) | p-value | Unadjusted HR (95% CI) | p-value |
| SM Volume per SD increase | 0.98 (0.92 – 1.04) | 0.511 | **0.92 (0.88 – 0.97)** | **0.001** |
| SM Density per SD increase | **0.95 (0.93 – 0.98)** | **<0.001** | **0.84 (0.80 – 0.89)** | **<0.001** |
| Bone Volume per SD increase | **1.13 (1.07 – 1.20)** | **<0.001** | **1.04 (1.00 – 1.09)** | **0.042** |
| Bone Density per SD increase | **0.85 (0.80 – 0.91)** | **<0.001** | **0.86 (0.83 – 0.90)** | **<0.001** |
| SAT Volume per SD increase | **0.91 (0.86 – 0.97)** | **0.002** | **0.82 (0.78 – 0.86)** | **<0.001** |
| SAT Density per SD increase | **1.22 (1.15 – 1.29)** | **<0.001** | **1.34 (1.28 – 1.39)** | **<0.001** |
| EAT Volume per SD increase | **1.08 (1.04 – 1.13)** | **<0.001** | 1.02 (0.98 – 1.06) | 0.328 |
| EAT Density per SD increase | 1.04 (0.97 – 1.11) | 0.229 | **1.11 (1.06 – 1.16)** | **<0.001** |
| VAT Volume per SD increase | 1.05 (0.98 – 1.12) | 0.140 | 0.96 (0.92 – 1.00) | 0.071 |
| VAT Density per SD increase | **1.29 (1.23 – 1.35)** | **<0.001** | **1.51 (1.45 – 1.58)** | **<0.001** |
| IMAT Volume per SD increase | 1.04 (0.99 – 1.10) | 0.082 | 1.04 (0.99 – 1.09) | 0.106 |
| IMAT Density per SD increase | **1.24 (1.16 – 1.33)** | **<0.001** | **1.34 (1.27 – 1.41)** | **<0.001** |
| Multivariable analyses | | | | |
|  | Patients < 65 years (N = 4093) | | Patients ≥ 65 years (N = 5992) | |
|  | Adjusted HR (95% CI) | p-value | Adjusted HR (95% CI) | p-value |
| **SM Volume per SD increase** | **0.89 (0.83 - 0.95)** | **0.001** | **0.91 (0.87 – 0.97)** | **0.001** |
| **SM Density per SD increase** | **0.94 (0.9 – 0.97)** | **<0.001** | **0.90 (0.84 – 0.96)** | **0.002** |
| Bone Volume per SD increase | 0.93 (0.85 – 1.01) | 0.069 | **0.85 (0.80 – 0.90)** | **<0.001** |
| **Bone Density per SD increase** | **0.91 (0.85 – 0.97)** | **0.003** | **0.86 (0.82 – 0.90)** | **<0.001** |
| SAT Volume per SD increase | 0.94 (0.85 – 1.04) | 0.233 | **0.88 (0.82 – 0.95)** | **0.001** |
| **SAT Density per SD increase** | **1.19 (1.11 – 1.27)** | **<0.001** | **1.22 (1.17 – 1.28)** | **<0.001** |
| EAT Volume per SD increase | 1.04 (0.97 – 1.11) | 0.262 | 1.01 (0.96 – 1.05) | 0.790 |
| EAT Density per SD increase | 1.06 (0.99 – 1.14) | 0.076 | **1.10 (1.05 – 1.16)** | **<0.001** |
| VAT Volume per SD increase | **0.91 (0.83 – 0.98)** | **0.021** | **0.93 (0.88 – 0.98)** | **0.005** |
| VAT Density per SD increase | **1.18 (1.11 – 1.26)** | **<0.001** | **1.27 (1.21 – 1.32)** | **<0.001** |
| IMAT Volume per SD increase | **0.92 (0.86 – 0.98)** | **0.014** | 0.97 (0.91 – 1.02) | 0.209 |
| IMAT Density per SD increase | **1.24 (1.16 – 1.33)** | **<0.001** | **1.25 (1.18 – 1.32)** | **<0.001** |

**Supplementary Table 7:** Associations with death or myocardial infarction stratified by patient age. Hazard ratios (HR) reflect the risk per standard deviation (SD) increase. The multivariable model included age, sex, body mass index, medical history, coronary artery calcium, stress total perfusion deficit, stress left ventricular ejection fraction, change in ejection fraction, myocardial flow reserve, and revascularization within 90 days with each body composition component assessed separately. Significant associations in bold. CI – confidence interval, EAT – epicardial adipose tissue, IMAT – intermuscular adipose tissue, SAT – subcutaneous adipose tissue, SM – skeletal muscle, VAT – visceral adipose tissue.

Supplementary Table 8

| Death (n=2896) | | | | |
| --- | --- | --- | --- | --- |
|  | Unadjusted HR (95% CI) | p-value | Adjusted HR (95% CI) | p-value |
| SM Volume per SD increase | **0.88 (0.85 – 0.91)** | **<0.001** | 0.96 (0.92 – 1.00) | 0.071 |
| SM Density per SD increase | **0.93 (0.92 – 0.95)** | **<0.001** | 0.94 (0.91 – 1.98) | 0.001 |
| Bone Volume per SD increase | **1.17 (1.13 – 1.21)** | **<0.001** | **0.92 (0.88 – 0.97)** | **0.002** |
| Bone Density per SD increase | **0.77 (0.74 – 0.80)** | **<0.001** | **0.86 (0.83 – 0.90)** | **<0.001** |
| SAT Volume per SD increase | **0.80 (0.77 – 0.84)** | **<0.001** | **0.92 (0.86 – 0.98)** | **0.009** |
| SAT Density per SD increase | **1.33 (1.29 – 1.37)** | **<0.001** | **1.23 (1.18 – 1.29)** | **<0.001** |
| EAT Volume per SD increase | **1.07 (1.04 – 1.09)** | **<0.001** | 1.03 (0.99 – 1.07) | 0.143 |
| EAT Density per SD increase | **1.06 (1.02 – 1.10)** | **0.006** | **1.12 (1.07 – 1.17)** | **<0.001** |
| VAT Volume per SD increase | 1.03 (0.99 – 1.07) | 0.107 | **0.93 (0.89 – 0.98)** | **0.004** |
| VAT Density per SD increase | **1.46 (1.43 – 1.50)** | **<0.001** | **1.32 (1.27 – 1.37)** | **<0.001** |
| IMAT Volume per SD increase | **1.08 (1.05 – 1.12)** | **<0.001** | **1.05 (1.01 – 1.10)** | **0.018** |
| IMAT Density per SD increase | **1.38 (1.32 – 1.44)** | **<0.001** | **1.28 (1.22 – 1.35)** | **<0.001** |
| Myocardial infarction (n=690) | | | | |
|  | Unadjusted HR (95% CI) | p-value | Adjusted HR (95% CI) | p-value |
| SM Volume per SD increase | **0.90 (0.84 – 0.96)** | **0.003** | **0.78 (0.71 – 0.86)** | **<0.001** |
| SM Density per SD increase | **0.93 (0.90 – 0.97)** | **<0.001** | **0.92 (0.88 – 0.95)** | **<0.001** |
| Bone Volume per SD increase | **0.91 (0.85 – 0.97)** | **0.004** | **0.73 (0.65 – 0.81)** | **<0.001** |
| Bone Density per SD increase | 1.01 (0.95 – 1.08) | 0.891 | 0.98 (0.91 – 1.06) | 0.685 |
| SAT Volume per SD increase | **1.06 (0.99 – 1.13)** | **0.119** | **0.88 (0.78 – 1.00)** | **0.049** |
| SAT Density per SD increase | 1.02 (0.95 – 1.08) | 0.613 | **1.13 (1.03 – 1.24)** | **0.010** |
| EAT Volume per SD increase | **1.07 (1.03 – 1.11)** | **<0.001** | 0.94 (0.85 – 1.03) | 0.196 |
| EAT Density per SD increase | 0.95 (0.90 – 1.01) | 0.080 | 1.05 (0.96 – 1.14) | 0.268 |
| VAT Volume per SD increase | **1.11 (1.04 – 1.17)** | **0.001** | **0.89 (0.81 – 0.98)** | **0.016** |
| VAT Density per SD increase | **0.93 (0.87 – 0.99)** | **0.027** | **0.90 (0.83 – 0.98)** | **0.021** |
| IMAT Volume per SD increase | **0.88 (0.81 – 0.95)** | **0.001** | **0.67 (0.60 – 0.74)** | **<0.001** |
| IMAT Density per SD increase | 0.99 (0.94 – 1.05) | 0.734 | **1.12 (1.03 – 1.23)** | **0.013** |
| Cardiovascular Death (n=737) | | | | |
|  | Unadjusted HR (95% CI) | p-value | Adjusted HR (95% CI) | p-value |
| SM Volume per SD increase | **0.92 (0.85 – 0.99)** | **0.028** | 0.96 (0.88 – 1.06) | 0.420 |
| SM Density per SD increase | 0.98 (0.82 – 1.05) | 0.537 | **1.13 (1.01 – 1.25)** | **0.029** |
| Bone Volume per SD increase | 1.06 (0.99 – 1.14) | 0.097 | **0.76 (0.68 – 0.85)** | **<0.001** |
| Bone Density per SD increase | **0.79 (0.73 – 0.85)** | **<0.001** | **0.88 (0.82 – 0.95)** | **0.002** |
| SAT Volume per SD increase | **0.79 (0.73 – 0.85)** | **<0.001** | **0.79 (0.70 – 0.90)** | **<0.001** |
| SAT Density per SD increase | **1.42 (1.33 – 1.51)** | **<0.001** | **1.38 (1.28 – 1.50)** | **<0.001** |
| EAT Volume per SD increase | **1.08 (1.04 – 1.13)** | **<0.001** | 1.04 (0.97 – 1.12) | 0.304 |
| EAT Density per SD increase | **1.11 (1.03 – 1.20)** | **0.010** | **1.18 (1.08 – 1.28)** | **<0.001** |
| VAT Volume per SD increase | 0.98 (0.91 – 1.05) | 0.553 | **0.83 (0.75 – 0.92)** | **<0.001** |
| VAT Density per SD increase | **1.44 (1.37 – 1.51)** | **<0.001** | **1.28 (1.18 – 1.38)** | **<0.001** |
| IMAT Volume per SD increase | 0.96 (0.89 – 1.04) | 0.346 | **0.87 (0.79 – 0.97)** | **0.008** |
| IMAT Density per SD increase | **1.54 (1.41 – 1.67)** | **<0.001** | **1.47 (1.34 – 1.62)** | **<0.001** |
| Cardiovascular or Undetermined Cause of Death (n=1730) | | | | |
|  | Unadjusted HR (95% CI) | p-value | Adjusted HR (95% CI) | p-value |
| SM Volume per SD increase | **0.85 (0.80 – 0.89)** | **<0.001** | **0.92 (0.87 – 0.98)** | **0.009** |
| SM Density per SD increase | **0.92 (0.91 – 0.94)** | **<0.001** | **0.90 (0.88 – 0.93)** | **<0.001** |
| Bone Volume per SD increase | **1.31 (1.26 – 1.37)** | **<0.001** | 1.02 (0.96 – 1.09) | 0.507 |
| Bone Density per SD increase | **0.77 (0.73 – 0.81)** | **<0.001** | **0.87 (0.82 – 0.91)** | **<0.001** |
| SAT Volume per SD increase | **0.80 (0.76 – 0.85)** | **<0.001** | **1.12 (1.03 – 1.22)** | **0.007** |
| SAT Density per SD increase | **1.24 (1.18 – 1.30)** | **<0.001** | **1.07 (1.01 – 1.13)** | **0.019** |
| EAT Volume per SD increase | **1.08 (1.05 – 1.11)** | **<0.001** | 1.07 (1.02 – 1.11) | 0.002 |
| EAT Density per SD increase | 0.96 (0.92 – 1.01) | 0.092 | 1.03 (0.98 – 1.09) | 0.300 |
| VAT Volume per SD increase | **1.13 (1.08 – 1.18)** | **<0.001** | 1.06 (1.00 – 1.12) | 0.061 |
| VAT Density per SD increase | **1.46 (1.42 – 1.51)** | **<0.001** | **1.26 (1.20 – 1.33)** | **<0.001** |
| IMAT Volume per SD increase | **1.17 (1.13 – 1.22)** | **<0.001** | **1.18 (1.12 – 1.24)** | **<0.001** |
| IMAT Density per SD increase | **1.19 (1.13 – 1.26)** | **<0.001** | **1.08 (1.01 – 1.14)** | **0.024** |

**Supplementary Table 8:** Associations with individual event types for elevated values of tissue volume and density. Hazard ratios (HR) reflect the risk for each standard deviation (SD) increase in values. The multivariable model included age, sex, body mass index, medical history, coronary artery calcium, stress total perfusion deficit, stress left ventricular ejection fraction, change in ejection fraction, myocardial flow reserve, and revascularization within 90 days and each body composition component was evaluated separately. Significant associations in bold. CI – confidence interval, EAT – epicardial adipose tissue, IMAT – intermuscular adipose tissue, SAT – subcutaneous adipose tissue, SM – skeletal muscle, VAT – visceral adipose tissue.

Supplementary Table 9

|  | Unadjusted HR  (95% CI) | p-value | Adjusted HR  (95% CI) | p-value | Increase in  LR chi-square |
| --- | --- | --- | --- | --- | --- |
| SM indexed to BSA | **0.88 (0.85 – 0.91)** | **<0.001** | **0.91 (0.87 – 0.95)** | **<0.001** | **19.1** |
| SM % thoracic volume | **0.93 (0.90 – 0.97)** | **<0.001** | 0.98 (0.94 – 1.03) | 0.482 | 0.50 |
| Bone indexed to BSA | **1.12 (1.08 – 1.16)** | **<0.001** | **0.87 (0.83 – 0.92)** | **<0.001** | **30.3** |
| Bone % thoracic volume | **1.20 (1.16 – 1.24)** | **<0.001** | 1.02 (0.96 – 1.08) | 0.521 | 0.4 |
| SAT indexed to BSA | **0.84 (0.81 – 0.87)** | **<0.001** | **0.89 (0.84 – 0.94)** | **<0.001** | **15.0** |
| SAT % thoracic volume | **0.83 (0.80 – 0.86)** | **<0.001** | **0.91 (0.85 – 0.96)** | **0.002** | **9.5** |
| EAT indexed to BSA | **1.07 (1.04 – 1.09)** | **<0.001** | 1.02 (0.98 – 1.05) | 0.530 | 0.4 |
| EAT % thoracic volume | **1.10 (1.07 – 1.13)** | **<0.001** | 1.03 (1.00 – 1.07) | 0.063 | 3.3 |
| VAT indexed to BSA | **1.04 (1.01 – 1.08)** | **0.022** | **0.91 (0.87 – 0.95)** | **<0.001** | **17.5** |
| VAT % thoracic volume | **1.09 (1.06 – 1.13)** | **<0.001** | **0.95 (0.91 – 0.99)** | **0.013** | **6.3** |
| IMAT indexed to BSA | **1.04 (1.01 – 1.08)** | **0.009** | **0.95 (0.91 – 0.99)** | **0.019** | **5.7** |
| IMAT % thoracic volume | **1.09 (1.06 – 1.13)** | **<0.001** | 0.99 (0.95 – 1.03) | 0.495 | 0.5 |

**Supplementary Table 9:** Associations with death or myocardial infarction for volumes indexed to body surface area (BSA) or expressed as a percentage (%) of total thoracic volume. Hazard ratios (HR) reflect the risk per standard deviation (SD) increase. The multivariable model included age, sex, body mass index, medical history, coronary artery calcium, stress total perfusion deficit, stress left ventricular ejection fraction, change in ejection fraction, myocardial flow reserve, and revascularization within 90 days with each body composition component assessed separately. Significant associations in bold. CI – confidence interval, EAT – epicardial adipose tissue, LR – likelihood ratio, IMAT – intermuscular adipose tissue, SAT – subcutaneous adipose tissue, SM – skeletal muscle, VAT – visceral adipose tissue.

Supplementary Table 10

|  | Adjusted HR | P-value |
| --- | --- | --- |
| **SM Volume index (per SD increase)** | 0.82 (0.77 - 0.88) | <0.001 |
| **SM Density (per SD increase)** | 0.95 (0.90 - 1.02) | 0.148 |
| Bone Volume index (per SD increase) | 0.98 (0.91 - 1.04) | 0.457 |
| **Bone Density (per SD increase)** | 0.91 (0.87 - 0.95) | <0.001 |
| SAT Volume index (per SD increase) | 1.08 (1.00 - 1.17) | 0.051 |
| **SAT Density (per SD increase)** | 1.29 (1.21 - 1.38) | <0.001 |
| **EAT Volume index (per SD increase)** | 1.05 (1.01 - 1.09) | 0.011 |
| EAT Density (per SD increase) | 1.03 (0.98 - 1.07) | 0.269 |
| VAT Volume index (per SD increase) | 1.00 (0.94 - 1.07) | 0.914 |
| **VAT Density (per SD increase)** | 1.14 (1.09 - 1.19) | <0.001 |
| **IMAT Volume index (per SD increase)** | 1.11 (1.04 - 1.18) | 0.002 |
| IMAT Density (per SD increase) | 1.08 (1.01 - 1.16) | 0.018 |
| **Age (per SD increase)** | 1.26 (1.20 - 1.33) | <0.001 |
| **Male** | 1.24 (1.10 - 1.40) | <0.001 |
| Body mass index (per SD increase) | 0.99 (0.93 - 1.06) | 0.838 |
| **Hypertension** | 1.16 (1.05 - 1.29) | 0.005 |
| **Diabetes** | 1.29 (1.20 - 1.39) | <0.001 |
| Dyslipidemia | 0.92 (0.85 – 1.00) | 0.046 |
| Family History | 0.95 (0.87 - 1.05) | 0.314 |
| **Smoking** | 1.22 (1.08 - 1.39) | 0.002 |
| **Heart Failure** | 1.19 (1.09 - 1.30) | <0.001 |
| **Cancer** | 1.20 (1.09 - 1.32) | <0.001 |
| **Past myocardial infarction** | 1.31 (1.20 - 1.43) | <0.001 |
| Previous revascularization | 0.97 (0.89 - 1.06) | 0.532 |
| CAC 0 | Reference | - |
| **CAC 1-100** | 1.34 (1.18 - 1.51) | <0.001 |
| **CAC 101-400** | 1.37 (1.21 - 1.55) | <0.001 |
| **CAC > 400** | 1.52 (1.35 - 1.72) | <0.001 |
| **Log stress total perfusion deficit + 1 (per SD increase)** | 1.17 (1.11 - 1.22) | <0.001 |
| **Log Stress LVEF (per SD increase)** | 0.85 (0.82 - 0.88) | <0.001 |
| Change in LVEF (per SD increase) | 0.99 (0.95 - 1.02) | 0.54 |
| **Log Myocardial flow reserve (per SD increase)** | 0.81 (0.77 - 0.84) | <0.001 |
| Early revascularization | 0.95 (0.85 - 1.07) | 0.405 |

**Supplementary Table 10:** Multivariable model. Associations with death or myocardial infarction with body tissue components. Significant associations in bold. Overall model C-statistic was 0.787 (95% confidence interval 0.777 – 0.797). CAC – coronary artery calcium, DL – deep learning, EAT – epicardial adipose tissue, HR – hazard ratio, IMAT – intermuscular adipose tissue, LVEF – left ventricular ejection fraction, SAT – subcutaneous adipose tissue, SD – standard deviation, SM – skeletal muscle, VAT – visceral adipose tissue.
